# Voices from the front line: Community health workers’ perspectives on effectiveness and needs in Madagascar’s tuberculosis response

**DOI:** 10.1371/journal.pgph.0005797

**Published:** 2026-10-22

**Authors:** Onja Gabrielle Ravololohanitra, Alphonsine Razanamiarana, Michel Tiaray Harison, Justine Nafeno, Lydia Raveloarisoa, Harizaka Emmanuel Andriamasy, Haingotiana Razafimanantsoa, Anne Neumann, Nadine Muller

**Affiliations:** 1 Monitoring, Evaluation, and Research, Doctors for Madagascar, Antananarivo, Madagascar; 2 Global Digital Last Mile Health Lab at Charité Center for Global Health, Charité - University Medical Center Berlin, corporate member of Freie Universität Berlin and Humboldt-Universität zu Berlin, Berlin, Germany; 3 Community Health Worker, Antananarivo, Madagascar; 4 Programme National de Lutte contre la Tuberculose, Antananarivo, Madagascar; 5 Community Health Worker, Ampanihy, Madagascar; 6 Speciality Network: Infectious Diseases and Respiratory Medicine, Charité - University Medical Center Berlin, corporate member of Freie Universität Berlin and Humboldt-Universität zu Berlin, Berlin, Germany; 7 Berlin Institute of Health at Charité - University Medical Center Berlin, Berlin, Germany; ICDDR: International Centre for Diarrhoeal Disease Research Bangladesh, BANGLADESH

## Abstract

Community health workers (CHWs) are central to primary health care delivery in Madagascar, yet their perspectives on what enables effective work are rarely centered in program design and implementation. This study aims to examine the lived experiences of Malagasy CHWs engaged in tuberculosis (TB) program activities and to develop context-specific recommendations to strengthen their effectiveness in Madagascar’s TB response. We conducted a qualitative study nested within the formative phase of a participatory action research initiative aimed at strengthening Madagascar’s community health workforce. TB program support was used as a tracer condition for universal health coverage-relevant service delivery. Five focus group discussions were held with 26 CHWs in August-September 2024 in two contrasting regions (Analamanga and Atsimo-Andrefana). Discussions were conducted in Malagasy and its dialects, audio-recorded, transcribed, translated into French, and analyzed using inductive reflexive thematic analysis. CHWs articulated five interlinked priorities for effectiveness in the TB response: (1) stable and equitable socio-economic protection; (2) standardized, continuous, and practical training; (3) formative and collaborative supervision; (4) reliable provision of tools, equipment, transport, and personal protective equipment; and (5) recognition and integration within communities and the health system. CHWs emphasized reliability and fulfillment of promised entitlements and described fragmentation across implementing partners as a key source of inequity, uncertainty, and demotivation. CHWs’ accounts point to a persistent implementation gap between policy commitments and the reliability of day-to-day support. Addressing fragmentation and strengthening coordinated national stewardship and partner accountability will be essential to ensure predictable, equitable support for CHWs - a prerequisite for advancing equitable progress toward universal health coverage in Madagascar.

## Introduction

Community health workers (CHWs) provide essential primary health care in many low- and middle-income countries, particularly across sub-Saharan Africa. Their close ties to communities, socially and geographically, support health promotion, early disease detection, adherence support, timely referral, and long-term follow-up, thereby improving the availability, accessibility, acceptability, and quality of health services [1].

Global momentum increasingly recognizes CHWs not only as programmatic assets but also as rights-holders whose work must be protected. The 2018 Declaration of Astana [2] and WHO’s guideline on optimizing CHW programs [3] position CHWs at the center of primary health care and specify obligations for fair remuneration, training, supportive supervision, and adequate supplies. Evidence from Brazil, Ethiopia, and Nepal [4] suggests that when CHWs are integrated with clear roles, equitable pay, and non-punitive supervision, progress towards universal health coverage (UHC) accelerates [5]. The 2023 Monrovia Call to Action urges governments and partners to fully fund, integrate, and safeguard CHWs [6]. Yet despite this momentum - and the growing visibility of advocacy platforms that amplify CHW voices - CHWs’ participation in decision-making and program design often remains limited [7]. Consequently, in many settings, CHWs continue to face precarious or opaque compensation, uneven access to training and tools, limited opportunities for participation and accountability, and weak recognition within decision-making structures [8–10].

Madagascar, an island nation of about 32 million people [11], faces substantial primary health-care delivery challenges, with a UHC service coverage index of 35 out of 100 in 2019 [12]. Nearly 59% of the population lives in rural areas [13], where CHWs play an essential role in extending access to care and advancing progress towards UHC. CHWs are officially recognised in national policy, including the *Politique Nationale de Santé Communautaire* (PNSC), and largely operate on a voluntary basis [14]. Program implementation involves multiple partners, contributing to fragmentation across health topics and gaps in accountability. In this plural landscape, CHWs may be uncertain about their entitlements (e.g., compensation, training, equipment, transport), and the CHW workforce is seldom systematically involved in program design and evaluation. As a result, CHWs’ own accounts of what strengthens (or constrains) their effectiveness are rarely centered, limiting evidence on what they receive in practice and whether support programs adequately enable them to work effectively.

Tuberculosis (TB) remains a major public health challenge in Madagascar. The estimated TB incidence has remained largely unchanged at 233 cases per 100,000 population since 2013 [15]. Around 40% of children under five years of age are stunted, indicating high rates of undernutrition among children, a major risk factor for developing TB [16]. Estimates indicate that only about half of incident TB cases are notified and treated annually, pointing to substantial gaps in case detection and access to care [17]. The southern regions of the country, including the Grand Sud, bear a disproportionate burden, with notification rates substantially higher than in the central highlands [18]. Madagascar's National TB Programme (Programme National de Lutte contre la Tuberculose, PNLT), established in 1991 and predominantly financed through international donors, coordinates TB care through a country-wide network of public, faith-based, and private diagnostic and treatment centres [17]. CHWs are positioned as the community-level arm of the programme, contributing to sensitisation, presumptive case identification, referral, treatment adherence support, and household contact tracing [14].

Despite their central role in delivering essential health services, including TB, in Madagascar, limited evidence captures CHWs’ own perspectives on the challenges they face and the support they require to work effectively within the health system. This study therefore examines the lived experiences and perceived constraints of Malagasy CHWs involved in the TB program support and identifies context-specific recommendations to strengthen their effectiveness. TB program support was used as a tracer condition for UHC because engagement in TB places particular demands on CHWs, including sustained patient follow-up, infection control, stigma-sensitive communication, and reliable referral pathways. These features intensify the need for consistent support and effective governance, thereby exposing gaps that may be less visible in less stigmatized or more episodic areas of care. Drawing on CHWs’ perspectives, we analyze systemic barriers and opportunities for improvement. The work forms part of a broader intersectoral action research initiative aimed at strengthening Madagascar’s community health workforce [19].

## Methods

### Ethics statement

The study was approved by the Comité d'évaluation éthique pour la recherche biomédicale (CEER) at the National Public Health Institute (Institut National de la Santé Publique et Communautaire, INSPC) in Madagascar (003/2023_CEER INSPC/IRB) and by the Ethics Committee of Charité - Universitätsmedizin Berlin in Germany (EA2/083/23). Oral and written informed consent were obtained from all participants. Anonymity was maintained at all stages of the research, and all data were de-identified prior to translation. Audio recordings and transcripts were anonymized and assigned unique codes before being securely stored in a restricted-access drive, accessible only to members of the research team.

### Study design

We conducted a qualitative study nested within the formative phase of a participatory, intersectoral action research initiative aimed at strengthening Madagascar’s community health workforce [19]. CHWs were engaged as partners in identifying challenges, generating evidence, and interpreting findings to inform practice-oriented improvements. The findings were subsequently presented at a design-thinking workshop held in March 2025 in Analamanga region, convening CHWs and policy makers, where participants jointly validated the identified priorities and defined actionable measures for strengthening the CHW workforce.

To anchor the analysis in specific service delivery realities while examining broader system enablers and barriers, we used TB as a tracer condition for two reasons. First, Madagascar’s persistently high and stagnant TB burden [15] makes effective community-based TB service delivery a national necessity. Second, TB combines several features that make it analytically demanding and therefore well suited as a tracer of broader CHW system support: it requires sustained, multi-touchpoint engagement (sensitisation, screening, referral, treatment adherence support, and contact tracing); it is highly stigma-sensitive; and it depends on reliable infection prevention measures and equipment. Altogether, these features place high demands on the structural support that characterise CHW programmes, and suggest that weaknesses in any of these dimensions become visible in CHWs’ lived experiences of TB-related work.

### Study setting

Madagascar is an island nation in the Indian Ocean. An estimated 80% of the population lived below the international poverty line (<US$2.15/day, 2017 purchasing power parity) between 2022 and 2024 [20]. The economy is based mainly on subsistence farming, livestock, fishing, and the informal sector. The health system is organized in a pyramid with Basic Health Centers (Centres de Santé de Base - CSB) at community level, district hospitals, and regional or central referral hospitals. A national network of approximately 50,700 volunteer CHWs serves on the front line [21], providing prevention, screening, and patient support and linking communities to CSBs. Under national policy, CHWs are volunteer, community-selected lay health workers without formal education requirements; basic literacy and community acceptance are typically considered for selection, and training varies across the Ministry of Public Health and partner-supported programmes. National policy documents outline the overarching context and working conditions, such as the national strategic plan for strengthening community health [14] and the Community Activities Package guide (Guide PAC) [22].

This study was conducted in two epidemiologically, socioeconomically, and geographically contrasting regions. Analamanga, which includes the capital Antananarivo, is largely urban and peri-urban, with high population density, relatively higher service coverage, and a TB notification rate of 176 per 100,000 in 2024 [18]. Atsimo-Andrefana, the country’s largest administrative region in the southwest, is predominantly rural, with low population density, sparse health infrastructure, and high multidimensional poverty [11]. The TB notification rate in this region was 313 per 100,000 in 2024 [18]. Access to care in this region is largely constrained by distance, transport, and household economic barriers [23]. Including CHWs from both regions was a purposive decision to capture diversity of operating conditions and equity gaps that shape CHWs’ engagement in TB-related services.

### Sampling and selection of participants

Participants were recruited using a two-stage purposive sampling strategy. In the first stage, CSBs were selected in each region based on (i) TB activity during the preceding 12 months, (ii) catchment profile (urban, peri-urban, or rural), and (iii) logistical accessibility. In the second stage, CHWs from the selected CSBs were invited to participate if they were (1) aged 18 years or older, (2) currently active in their role, and (3) engaged in the TB response within the past 12 months. CHWs were excluded if they had less than 12 months of experience in their role, were not officially selected by their community based on local practices (including identification by their communities and validation by the head of the selected health centers), or declined to provide informed consent. Recruitment and discussion-group composition intentionally balanced gender, tenure, role, and catchment profile to capture a wealth of perspectives and field realities. This distribution reflects the composition of the active CHW workforce at the selected health centers in each region, as confirmed with local health facility staff prior to recruitment. Detailed sociodemographic characteristics such as level of education, years of experience, and training exposure were not systematically collected during data collection. All participating CHWs were volunteers without a formal salary. Participants were recruited between 19 August 2024 and 25 September 2024, during which time five focus group discussions (FGDs) were conducted.

### Data collection

FGDs were conducted in Malagasy and its respective dialects (Merina (highlands/Antananarivo), Mahafaly, Antandroy, and Masinkoro (Atsimo-Andrefana)) by a trained qualitative research team (coordinator, lead moderator, note taker). The data collection team had no prior relationship with participants. All moderators and note-takers were external to the communities and were not affiliated with local health facilities or community programs, reducing the risk of social desirability bias linked to familiarity. A local partner organization assisted with logistical arrangements and participant outreach but did not take part in data collection or analysis. The research team remained aware that participants might perceive researchers as linked to donor-supported health structures, potentially shaping how they expressed criticism or expectations. To reduce this influence, moderators emphasized the independence of the study and encouraged open discussion of both positive and negative experiences.

We used a semi-structured, thematic guide to explore CHW recruitment, roles, working conditions, training, incentives, supervision, professional identity, and factors influencing overall and TB-specific commitment. The guide was pilot-tested through two mock interviews with CHWs from outside the study area to assess clarity, relevance, flow, and duration. These participants were not included in the final study sample. The full focus group discussion guide is provided in S1 File. Participants were asked to propose concrete recommendations for improving CHW effectiveness, working conditions, and support and needs. Each discussion concluded with a co-design segment to generate practical, context-based recommendations for strengthening community health implementation. However, CHWs were not involved in data coding, formal analysis, or theme development, which remained researcher-led within a reflexive thematic analysis framework. The sessions lasted an average of 88 minutes.

Prior to each FGD, participants were informed about the study’s objectives, procedures, potential risks and benefits, confidentiality arrangements, and the voluntary nature of their participation. Written informed consent was obtained and then verbally reconfirmed at the beginning of each discussion. The focus group discussions were audio-recorded, transcribed verbatim, and translated into French with bilingual supervision to ensure that meaning and cultural nuances were preserved. In accordance with standard research practice in Madagascar, participants were reimbursed for their transportation costs.

### Data analysis

We analysed the French transcripts alongside Malagasy recordings through reflexive thematic analysis (TA) as outlined by Braun and Clarke [24,25]. The approach was selected for three reasons: it foregrounds researcher reflexivity within a constructivist-interpretivist paradigm; it accommodates an inductive, iterative analytic strategy unconstrained by prior theory, consistent with the study's exploratory and formative aim; and it supports the identification of latent as well as descriptive meaning, particularly relevant for deriving practice-oriented recommendations. The analysis was iterative, beginning with repeated readings of the transcripts to familiarise the team with the content, identify recurring ideas, and gradually develop a coding framework. As the analysis progressed, a dynamic codebook was created, renamed, merged, split, or adjusted as understanding deepened [24,25]. This flexibility allowed the categories to capture the diversity and nuance of participants’ accounts. The analysis process is illustrated in Fig 1.

**Fig 1:**
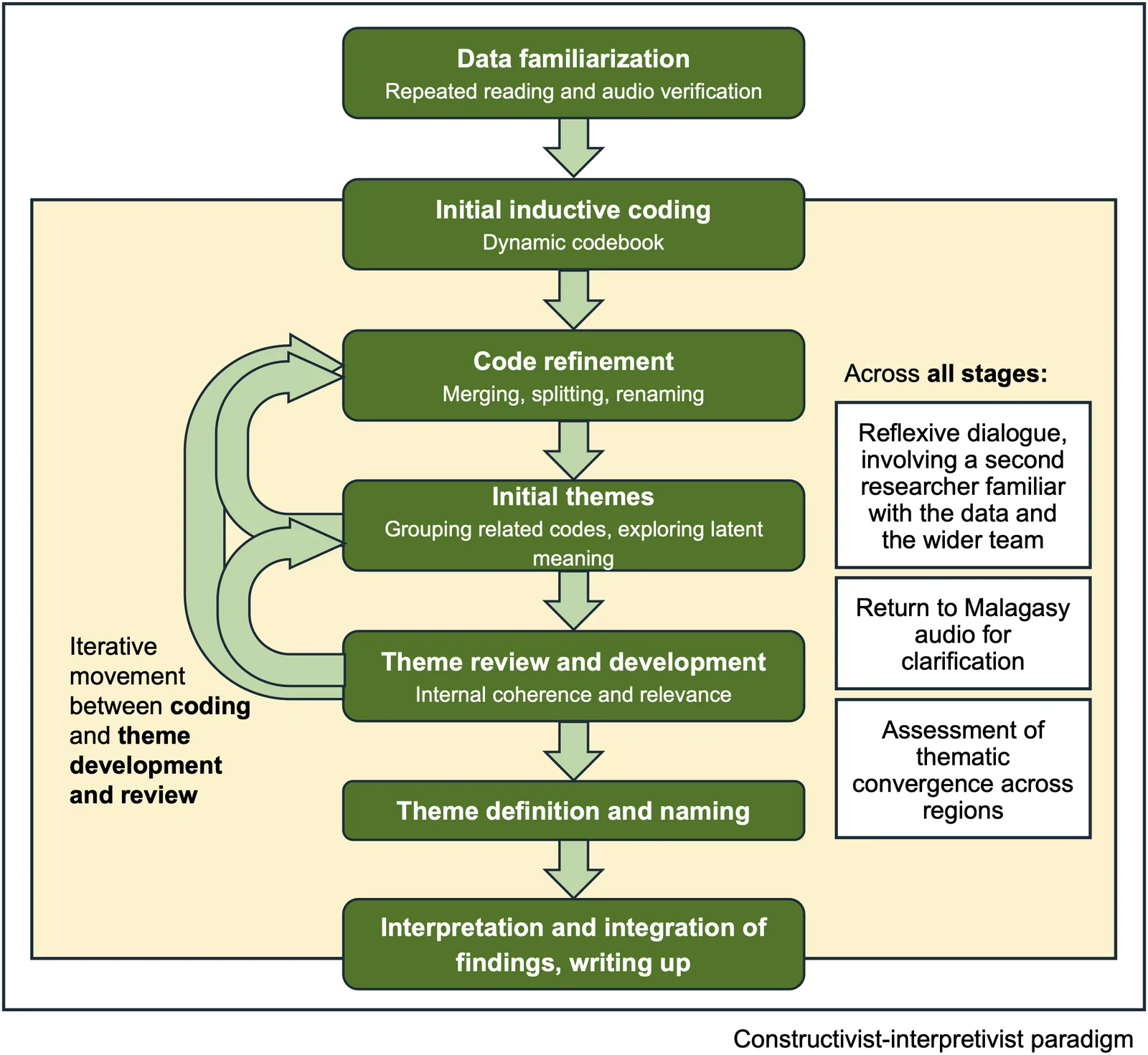
Iterative reflexive thematic analysis process used to examine community health workers’ perspectives.

The analysis was led by the first author, who managed coding and interpretation throughout. CHWs were not involved in formal coding or theme development; however, interpretive discussions during FGDs contributed to refining emerging understandings and informing practice-oriented recommendations. For ambiguous or complex segments, the analyst returned to the original-language transcripts and audio recordings before finalising codes. A second researcher reviewed selected excerpts and discussed code formulation, leveraging the team's differing proximity to CHWs’ lived realities as a reflexive resource in line with Braun and Clarke.

Code organisation, identification of relationships, and final structuring into themes and subthemes were conducted in NVivo (QSR International). Themes were defined by internal coherence, relevance to the research question, and grounding in participants’ narratives. Data adequacy was assessed in terms of depth, richness, and thematic convergence rather than numerical saturation. Recurring patterns and priorities were identified across the five FGDs, including among participants from both urban and rural settings. While some contextual differences emerged, particularly regarding access to training and material support, no substantially new thematic dimensions arose in the later discussions. The recurrence of key themes across two contrasting regions supported the sufficiency of the dataset for addressing the study objectives. Reporting follows the Consolidated Criteria for Reporting Qualitative Research (COREQ) checklist (S2 File).

## Results

A total of 26 CHWs participated in five FGDs: three in the Analamanga region and two in the Atsimo-Andrefana region. The participants’ characteristics are presented in Table 1.

**Table 1:** Participant characteristics for CHW focus group discussions, Madagascar, 2024. CHW = community health worker, TB = tuberculosis.

Characteristic	Category	Overall n=26 (%)	Analamanga n = 14	Atsimo-Andrefana n = 12
**Gender**	Female	16 (61.5)	13	3
	Male	10 (38.5)	1	9
**Age (years)**	Range	26-74	38 - 74	26 – 62
**CHW role**	Generalist	15 (57.7)	6	9
	TB-focused	11 (42.3)	8	3
**Area**	Urban	10 (38.5)	11	0
	Peri-urban	3 (11.5)	3	0
	Rural	13 (50.0)	0	12

The analysis generated five interlinked themes describing CHWs’ perceived needs to improve their effectiveness in Madagascar’s TB response: (1) stable and equitable socio-economic protection; (2) standardized, continuous training; (3) formative and collaborative supervision; (4) reliable tools, equipment, and transport; and (5) recognition and integration within the health system.

### Stable and equitable socio-economic protection

Across both regions, socio-economic protection emerged less as a request for income than as a marker of whether the system supporting CHWs could be trusted. Participants framed reliability, transparency, and equity in distribution as defining features of economic protection. CHWs consistently described the need for comprehensive and stable socio-economic protection to enable and sustain their engagement. They reported a dual source of frustration: inadequate compensation and a perceived failure to uphold promised terms:

*“There is a small fee for completing this report (monthly TB case activity report). You will receive your payment in a month. And the work is done, but the low pay doesn't match what was promised (by the program or supervisors).” (male CHW, rural Atsimo-Andrefana)*

CHWs also expressed a need for a basic level of economic security, enabling them to carry out their role with dignity, as well as formal recognition commensurate with their commitment. For several, the establishment of a professional status, including a regular salary and basic social protections, was deemed essential for long-term commitment. Others emphasized fixed, regular, and transparent compensation, whether as a salary, stipend, or allowance, as a matter of social justice, recognition, and minimum economic stability:

*“If we were given a small compensation of 100,000 Ar (22.5$) per month, it would be a way to make this task more acceptable to us, provided we had 100,000 Ar per month. This would be a change to implement.” (male CHW, rural Atsimo-Andrefana)*

The harmonised implementation guide for community health programmes in Madagascar [14] provides for financial incentives for CHWs. However, participants reported uneven implementation, with disparities in remuneration across actors and projects.

*“[...] We work as CHWs to accomplish tasks, and you (the NGOs) say that you (CHWs) will be helped by things like a telephone to communicate between us. So, for example, there are eight of us. And then only two of them get these things, even though they've all done the same job, which creates demotivation among us. The others get nothing, so it's just two people who benefit.” (male CHW, rural Atsimo-Andrefana)*

This perceived inequity was described as weakening cohesion and motivation among CHWs. Participants called for improved payment organization and transparency, including a clear display of amounts due, predictable schedules, and verification mechanisms.

Many also suggested performance-based incentives based on criteria they considered objective (such as number of TB cases referred, quality of patient follow-up, engagement in sensitization), while acknowledging that some indicators are harder to measure.

Finally, CHWs stressed the need for social support in the event of illness or serious personal events (e.g., accidents, pregnancy, family death):

*“When we're sick, we go to the CSB2 (community health center) to get medicine. We've already spoken directly to the general manager, but he replied that it was voluntary work and that he couldn't help us because it would become too much if they had to give us medicines. But sometimes we really need this little support, even for basic medicines, because sometimes we don't have the money to buy it, given that we do this work voluntarily and our income isn't even enough to buy medicines.”* (female CHW, urban Analamanga).

### Standardized, reality-grounded training

Training was discussed less as a technical input than as an indicator of system investment in the CHW workforce. Participants positioned training quality and continuity as a signal of how seriously decision-makers regarded CHWs as professional actors, and articulated a recurring tension between standardised expectations and uneven delivery. Within this framing, training was identified as a key lever to enhance effectiveness in the TB response. Many emphasised the need for ongoing, structured, and relevant training covering both technical knowledge and practical field skills. Regular refresher sessions were requested on TB-specific topics such as symptom detection, case management, adherence monitoring, and referral procedures. A single initial training was viewed as insufficient given evolving practices and operational challenges:

*“The training we receive is standard and very effective for us, but it needs to be reinforced. The last training session took place three years ago, and there should be new sessions to strengthen our capacity.”* (male CHW, rural Atsimo-Andrefana)

CHWs called for training that includes transversal and relational skills such as communication, handling resistance, and adapting messages to vulnerable groups given the role of stigma and fear in hindering case detection and treatment adherence:

*“We have to go through training before becoming CHWs, because we never know what the people around us might pass on to us. If we don’t receive training—especially in terms of health—we won’t know how to handle it. We may be good, but not as good as we think. That’s why training is essential.”* (female CHW, peri-urban Analamanga)

Another recurrent concern was perceived inequity in access to training, with opportunities varying widely by location and implementation partners, disfavouring specifically CHWs in rural and remote areas:

*“In our area, we've never had any refresher training. But we hear that in other communities, they have received new training on tuberculosis. Why not us?”* (male CHW, rural Atsimo-Andrefana)

Participants therefore insisted on nationally standardized training, with a shared base of knowledge and skills, tailored to local realities, such as limited mobility, poor digital access, and cultural differences in rural areas requiring adaptation of content and teaching methods:

*“Our suggestion is that you (the system) watch over us and support us. We frequently need training to strengthen our capacity and ensure that our knowledge is comprehensive.”* (female CHW, rural Atsimo-Andrefana)

They also stressed that training must be practical and grounded in reality, such as the use of daily reporting tools, field communication techniques adapted to the local context, concrete problem-solving, and simulated home visits.

### Formative and collaborative supervision

Supervision emerged as an indicator of how the formal health system perceived CHWs—as workers to be controlled or as colleagues to be supported. The theme captures a desired shift from a hierarchical-punitive model towards a collaborative, formative one anchored in mutual respect. CHWs reported perceiving the current supervision model as top-down or punitive and disconnected from the realities of their work. They expressed the desire for a shift from control-based approaches to more formative, supportive, and collaborative supervision focused on guidance rather than fault-finding or punishment:

*“[...] as soon as you leave at 8 a.m. for the appointment in the fokontany, there is already a supervisor waiting for you. They accompany you everywhere [...] around noon, they called us saying: ‘We have arrived to inspect your work.’ Then we visited all the households. We checked the child’s finger to see whether they had received the vaccine or not [...]”* (female CHW, urban Analamanga)

Participants specifically suggest regular, structured supervision based on dialogue, mutual respect and encouragement. A supportive approach – described as dynamic, motivating, and positive - was preferred:

*“Supervision shouldn't be frightening; it should enable us to get on with the job and move it forward. It's not there to scare us, but to ensure that we get the job done.”* (male CHW, rural Atsimo-Andrefana)

CHWs recommended simple, accessible tools adapted to their profiles (often low literacy levels, limited connectivity) to help monitor activities, track progress, and identify training needs - without adding unnecessary workload. Supervision was also seen as an extension of training that reinforces skills, adjusts practices, and flags areas for further learning:

*“Supervision isn't a bad thing; it's beneficial. It's what allows you to see the work that's not yet finished or that needs to be continued. [...] supervision allows you to understand your own work, to see where there are little mistakes and correct them, and to take your work further. It makes you evolve, you say to yourself, ‘ah, is this what it means?’”* (male CHW, rural Atsimo-Andrefana)

Several CHWs also proposed involving local leaders in certain aspects of supervision, particularly in sensitive areas like TB screening:

*“If the chef Fokontany (local village lead) knows what we're doing, he can support us. Otherwise, people ask us: who sent you? Why are you coming to my house?”* (female CHW, urban Analamanga)

Given the elevated stigma surrounding TB, participants noted that home visits, presumptive case investigations, and referrals can be poorly perceived by families, giving rise to resistance or fear:

*“Convincing the patient to agree to an analysis is really difficult. As my colleague said, for us Madagascans it's a shame to have the disease, and convincing them is still difficult, even to bring them here.”* (female CHW, urban Analamanga)

### Access to work tools, equipment, and transport

Participants framed access to tools, equipment, and transport as an enabler of professional dignity and community credibility: a properly equipped CHW was seen as more competent and worthy of community trust, while improvising or relying on personal resources was experienced as undermining both effectiveness and self-respect. During FGDs, a frequently cited obstacle was disrupted availability of essential tools and resources for carrying out duties. Participants emphasized that these prerequisites were viewed as needing systematic and permanent rather than exceptional provision. One female CHW explained:

*“We [...] should have a specific uniform as promoters, because ours, the old one, is in a bad shape, it's too old! It hasn’t been replaced in nearly 20 years, we've only had one since the beginning, and what's more, it's bad. For example, we work in summer! We need raincoats, and we also need something to carry the equipment we use to raise awareness about tuberculosis!”* (female CHW, peri-urban Analamanga)

The needs expressed fell into two main categories: Basic functional equipment and tools to improve feasibility and efficiency.

#### Basic functional equipment.

Participants reported that the absence of basic resources such as forms, registers, and health education materials (e.g., posters, brochures) prompted them to improvise or rely on personal resources, which they perceived as affecting quality, consistency, and credibility of their work. One female explained:

*“For materials too, when we go out into the field, they should be complete. You (the system) should also give materials to the CHWs to help them explain to the people they are sensitizing.”* (CHW, female, peri-urban area, Analamanga)

#### Tools to improve feasibility and efficiency.

Transport-related difficulties were particularly emphasized by rural CHWs, who described walking long distances to reach patients or health centers, which they experienced as physically demanding and financially costly. Without access to bicycles or travel allowances (called “solom-paladia” in Malagasy, meaning walking allowance), CHWs explained that they often had to cover transportation costs out of pocket. Some reported that when they could not afford transport, visits and activities were postponed or missed.

*“We CHWs, when we do VAD (visites à domicile; home visits) we go very far, within the fokontany (village). We got phones, bags for TB, we need bicycles because the settlements we go to are very far away.” (male CHW, rural Atsimo-Andrefana)*

This need for mobility, as expressed by the CHWs, is directly linked to the lack of financial protection, as outlined in the first dimension.

Provision of personal protective equipment was also cited as essential, particularly when in contact with people with TB. The absence of masks, gloves, or hand sanitizer exposes CHWs to risks and signals neglect compared with better-protected facility-based healthcare workers. One female CHW from urban Analamanga explained:

*“We have to buy our own masks to protect ourselves, because when we arrive at 5 am to give the medicine to the patients, the houses are still closed, the air is confined [...]. That's what made me hesitate to continue this work, but with the knowledge I've acquired, I tell myself that if everyone was afraid, the disease would spread even further.” (female CHW, urban Analamanga)*

Participants noted that tools and equipment availability shaped how they were perceived within their communities: a properly equipped CHW was described as more competent and credible. They emphasized that “equipment” also referred to symbolic or visual items, particularly campaign-specific uniforms, which help signal legitimacy and strengthen CHWs’ visibility during community activities. In contrast, they explained that lacking tools diminished their sense of professionalism and authority in the eyes of the community. One CHW explained:

*“It might be better to adapt the uniforms to the theme of the campaign to give more value to the work of the ACs (CHWs).” (female CHW, urban Analamanga)*

### Recognition and integration into the health system

Recognition was articulated not as a symbolic gesture but as a precondition for legitimacy; both within the formal health system and within the communities CHWs serve. Despite CHWs’ frontline role in the detection, awareness, and patient support, many reported feeling marginalized or seen as lower priority compared to formal healthcare workers:

*“It makes me think a bit and wonder whether the authorities really take ACs (CHWs) seriously. It's voluntary work, of course, but it's about time it was taken more seriously.” (female CHW, urban Analamanga)*

Participants indicated that this perceived lack of formal recognition had consequences for intrinsic motivation, legitimacy in the community, and collaboration with formal healthcare staff. Several described feelings of discouragement:

*“Sometimes we're disappointed because, [...], CHWs work hard for the community, yet if one of us dies or falls ill, nobody cares, yet if it's an artist who dies, he gets a medal. We've worked for decades, some have done the job for 40 years, but the state doesn't consider us.” (female CHW, urban Analamanga).*

Concerns were also raised about structural instability of community health programs, in which CHWs could be dismissed without clear justification:

*“What we insist on is that when the political leadership changes, we should not be pushed aside under the pretext that there are new people. We would not accept that. We are ready to continue as long as possible. It would be unfair to replace us with younger people or those close to local authorities, such as graduates, children of chefs fokontany, or others.” (female CHW, urban Analamanga)*

Many CHWs described institutional recognition as important for strengthening their credibility, clarifying roles, and fostering better collaboration with professional healthcare staff. They reported a desire to be treated as equal partners - with rights of their own - able to report on-the-ground realities, advocate for community needs, and participate actively in local health decision-making.

## Discussion

CHWs participating in this study across two regions identified five interlinked priorities for effective engagement in the TB response: basic financial protection, standardized and practical training, non-punitive supervision, reliable provision of tools and transport, and recognition within the community and the health system. These priorities were consistent across the two contrasting regions, suggesting that they are systemic rather than context-specific constraints in community health implementation.

From a UHC perspective, adequately supported and well-governed CHW programmes are essential for equitable service delivery. Despite persistently poor health indicators, minimal progress toward UHC [12], and the presence of a large CHW workforce in Madagascar, there is little peer-reviewed evidence on CHWs’ working conditions and the support they actually receive. While prior research has documented workload pressures and socioeconomic constraints [26], less attention has been given to how CHWs themselves experience the structural conditions shaping their work. We used TB as a tracer condition to examine how these structural factors operate in a demanding service area. Engagement in TB care requires sustained follow-up, infection prevention, and stigma-sensitive communication, and is therefore particularly sensitive to weaknesses in training, supervision, and material support [27]. Although some challenges, such as limited access to personal protective equipment, are specific to infectious disease contexts, most priorities identified by CHWs extend beyond TB programming and point to broader system-level governance weaknesses. We therefore anticipate that findings will be broadly transferable across community health domains in Madagascar, since most programmes share a similar set of structural support needs. However, given the marked fragmentation of community health implementation across actors and financing streams, this transferability requires confirmation through further domain-specific research.

While the identified five priorities are broadly consistent with the global CHW literature, our analysis extends current understanding in three respects: First, it shifts the focus from what support CHWs require to how accountability for that support is operationalised. Second, it demonstrates how programmatic fragmentation generates inequity within the CHW workforce itself. Third, it shows, from the workforce's own perspective, that a central tension is less between volunteerism and salaried employment than about credible governance configurations in the absence of either. Each is developed in turn below.

### Accountability for essential support

CHWs’ accounts in this study highlight that effectiveness depends not only on the formal provision of technical inputs but equally on the reliable and equitable delivery of support in practice. Their perspectives therefore extend beyond identifying practical needs to questioning whether governance arrangements ensure that agreed support is consistently implemented. CHWs stressed the importance of reliability and fulfilment of promised entitlements. This shifts the debate from *what* CHWs need (which is widely documented) to *how* accountability is operationalised, so that support is delivered with predictability and fairness. Testimonies describing unfulfilled commitments reveal a gap between formal expectations and lived reality, undermining trust, motivation, and engagement at the frontline. These findings align with evidence that CHW programme performance is shaped by relational and hierarchical dimensions, including recognition, respect, and the quality of relationships with both communities and the formal health system [28]. CHWs’ consistent emphasis on the gap between promised entitlements and lived support resonates with broader calls to treat CHWs as rights-holders and system actors, while underscoring that accountability must be understood as a condition for sustaining trust and motivation [29]. In practice, defining a minimum support package alone may be insufficient without clear mechanisms to ensure reliability, transparency, and equity in its implementation.

### Fragmentation and intra-workforce inequity

In Madagascar, domestic public financing for health remains low; public expenditure accounted for only 1.4% of GDP in 2020 [30]. As a result, the health system relies on external assistance, including major investments from the World Bank, Gavi, the Global Fund, and formerly USAID [31–33]. This externally financed landscape has led to a partner-driven implementation context in which CHWs described uneven remuneration, inconsistent access to training, and fragmented supervision across programs. Several CHWs participating in this study described working with multiple NGOs simultaneously, often without coordination or consistent social benefits. This reveals that fragmentation operates not only at the system level but also generates inequity within the CHW workforce itself. Participants described this as lacking transparency and fairness, and as fostering frustration and a sense of injustice. These observations resonate with broader evidence that fragmented programme architectures weaken standardisation, accountability, and workforce equity, thereby constraining CHW effectiveness [19,28,34]. Evidence also shows that per diem practices can distort priorities when daily allowances vary across funders and activities, incentivising CHWs in resource-constrained settings to prioritise higher-pay tasks and partners [35,36]. Even when equity-oriented approaches are implemented within individual projects, these efforts may not accumulate into a coherent national system. In Madagascar, fragmentation persists despite national efforts to harmonise community health implementation, including the Guide de mise en œuvre harmonisée du programme de santé communautaire (Guide MEO) [37], issued by the Ministry of Health in 2022, which seeks to standardise CHW roles, incentives, training, and supervision across partners. Participants’ accounts suggest that uneven implementation and limited accountability for this framework continue to undermine coordination and predictability of support.

### Governance configurations beyond the volunteer-salaried binary

Our findings also highlight tensions within Madagascar’s volunteer-based community health model: although volunteerism remains the formal policy framework, CHWs described irregular and fragmented support shaping their everyday reality. In this resource-limited setting, the absence of a basic safety net, even for CHWs’ own health needs, emerges as a central weakness of the model. Concurrent research shows that more than seven in ten CHW households live on less than US$30 per month [19], with other evidence describing heavy workloads and livelihood pressures [26]. Comparative research from 24 low-income countries demonstrates that remuneration models, whether fixed, performance-based, or hybrid, can enhance motivation, though irregular payments remain a key challenge [38]. Taken together, our findings suggest that the core tension is less in the volunteer status per se than in the absence of predictable material support and social protections. Importantly, CHWs in our study did not uniformly call for full salaried employment but emphasised reliable, equitable, and transparent minimum support. This challenges simplistic binaries between volunteerism and salaried professionalisation. In this context, professionalisation appears less as a contractual shift and more as a governance configuration grounded in reliability, fairness, and recognition that participants identified as preconditions for meaningful engagement.

### Implications for strengthening CHW programmes

Across these three threads, participants’ accounts converge on the need for strengthened governance and coordination across actors. Alignment of incentives, harmonisation of supervision practices, and shared accountability mechanisms may help reduce fragmentation, narrow the gap between policy commitments and frontline realities, and strengthen trust. Establishing a more effective national CHW program in similar contexts will likely depend on strengthened governance arrangements. Reflecting the multi-actor implementation landscape that participants described, formal stewardship rests with the Malagasy government, while both government services and implementing partners shape how consistently and equitably support is delivered in practice.

### Strengths and limitations

This study provides systematically analysed qualitative evidence foregrounding CHWs’ perspectives in Madagascar. Strengths include its participatory design embedded into a broader action research initiative, the inclusion of two contrasting regions, the use of local dialects to enable authentic participant expression, and the subsequent validation of findings with CHWs and policymakers through a joint workshop. Several limitations should be considered when interpreting the findings. First, the study was limited to five FGDs across two regions and may not capture the full diversity of CHW experiences nationwide. Second, the use of TB as a tracer condition facilitated concrete discussions of service delivery realities, including stigma, long-term follow-up, infection risk, and referral pathways, but may have emphasised priorities specific to infectious disease programmes and may not be fully generalisable across all community health activities. Third, the gender composition of participants reflects contextual workforce structures in urban and rural settings and may have shaped the perspectives expressed. Fourth, the analysis was led by a single coder, with researcher triangulation at key interpretive moments rather than full double-coding. Fifth, participants may have perceived the research team as linked to NGOs, donor-supported programmes, or health institutions, which could have influenced how they expressed criticism or expectations; the team remained attentive to this potential social desirability bias throughout the study. Despite these limitations, the system-level enablers identified here are consistent with findings from CHW programmes in other settings and were validated through subsequent stakeholder engagement.

## Conclusions

In conclusion, CHWs in Madagascar remain the cornerstone of community health delivery, yet report frequently operating without the predictable support, recognition, and material means required to fulfil their role effectively. Addressing gaps in coordination, reliability, and accountability across actors may be essential to narrowing the gap between policy commitments and frontline realities. Strengthening the CHW workforce is not only a matter of fairness but also a prerequisite for equitable progress towards UHC in Madagascar.

### Reflexivity and positionality

The two main authors brought different perspectives to this study. OR is a Malagasy social scientist working in community health at the NGO Doctors for Madagascar. Her familiarity with the local context and health system supported nuanced interpretation of participants’ accounts. NM is a clinician and global health researcher based in Europe and formerly served as a board member of Doctors for Madagascar. She is a research group lead at Charité – Universitätsmedizin Berlin. Her position allowed for critical distance in interpreting the findings. The two authors are aware that their social and institutional roles could influence data interpretation. Participants may have perceived the research team as linked to NGOs, donor-supported programmes, or health institutions, which could have shaped how they expressed criticism or expectations. The team therefore remained attentive to potential social desirability bias and power asymmetries throughout the study. Throughout the analysis, they held regular discussions to reflect on assumptions, question interpretations, and consider how their positions shaped the analytic process.

## Supporting information

S1 File Semi-structured focus group discussion topic guide. (PDF)

S2 File Completed Consolidated Criteria for Reporting Qualitative Research (COREQ) checklist. (PDF)
