# Supplementary material for: Voices from the front line: Community health workers’ perspectives on effectiveness and needs in Madagascar’s tuberculosis response
Source: PLOS Glob Public Health. 2026 Oct 22;6(10):e0005797. doi: 10.1371/journal.pgph.0005797 (PMC13690241; doi:10.1371/journal.pgph.0005797)
Supplement: Supplementary file 1 [file pgph.0005797.s001.pdf]

TOPIC Guide (original version)

Tari-dresaka amin'ny fanadihadiana lalina

1. Fampidirana
   - Fampahafantarana ny mapanadihady
   - Fampahafantarana mikasika ireo fomba fiasa tsara araka ny fenitra, anisan'izany ny fitanana tsiambaratelo
   - Fakàna ny fankatoavana
   - Fanadihadiana mombamomba ireo hadihadiana (mpisehatra iasana, taona, lahy sa vavy, sns)

2. Fifantenana mpanentana ara-pahasalamana

Lohahevitra

- Ny fahatongavana ho AC
- Ireo fepetra ilaina amin'ny fifantenana
- Ireo mpanapa-kevitra

Fanontaniana:

2.1. Azonareo lazaina ahy ve ireo toetoetran'ny atao hoe AC mahomby?

Hadihadio: *toetoetra itambaran'ny AC, fepetra na fahaizana takiana mahatonga AC*

2.2. Inona avy ireo dingana arahina ahatongavana ho AC? Inona avy ireo fepetra takiana?

Hadihadio: Ny fomba fifantenana AC; mitovy sa samihafa arakaraky ny inona: faritra na antony hafa? Iza avy no mamaritra ny fomba fifantenana AC? Manao ahoana ny fahitanao izay fomba fifantenana AC izay?

2.3. Iza no tena manapa-kevitra amin'ny fifantenana ho AC?

Hadihadio: ny fahitan'ny mpandray anjara ny olona manapa-kevitra

3. **Tontolo manodidina ny asa**

Lohahevitra:

- Ireo fahasarotana atrehina
- Ireo fanamorana
- Ny fahafioza-tena amin'ny maha AC, engagement

Fanontaniana:

3.1. Araka ny hevitrareo amin'ny maha AC anareo, manao ahoana ny tontolon'ny asa amin'ny ankapobeny?

Hadihadio: Fizotran'ny fiasa amin'ny ankapobeny: mora na sarotra, ny andraikitra sahanina, ny $

Hadiohadio:

3.2. **Inona no heverinareo fa zavatra manan-danja ilaina rehefa hanao ny asanareo?**

Hadihadio: Risi-po avy manosika anareo manokana, toy ny fankasitrahana, tambin'asa materialy

4. Iza ny Mpanaramaso anareo AC? ary manao ahoana ny toetoetra fanarahamaso misy amin'izao fotoana izao?

   4.1. Inona ny soso-kevitra nareo amin'izany?

5. <u>Ny hevitr'ireo AC momba azy sy ny mampirisika azy amin'ny asany</u>

   Ny lanjan'ny asan'ny AC eo amin'ny fiaraha-monina, eo amin'ny rafitra ara-pahasalamana

Fanontaniana:

5.1. Ahoana ny fahitanareo AC ny asa sahaninareo eo amin'ny rafitra ara-pahasalamana

Hadihadio: hatraiza ny maha zavadehibe ny AC, manao ahoana raha tsy misy AC

5.2. Ahoana ny fahatsapanareo ny andraikitra sahaninareo amin'ny maha AC eo anivon'ny fiaraha monina iasanareo?

Hadihadio: manolona ireo olona sahirana vao mahazo fitsabona, ireo lavitra tobim-pahasalamana, ireo marefo, ireo voailika

5.3. Manao ahoana ny fahafaham-po nareo manokana amin'ny tolotra omenareo ny firahamonina?

5.4. Araka ny fanadihadiana natao tamin'ny AC teo aloha: izy rehetra: 100 isan-jato no nilaza fa zava-dehibe hoan'ny fiarahamonina ny hiasanareo azy ireo an-tsitra-po, ahoana ny hevitrareo mikasika izany?

Hadihadio:

5.5. Inona no antony voalohany nanosika anareo hanao AC?

Hadihadio: mba tantarao aminay ny nahatonga anareo AC, inona ilay tena nandrisika

5.6. Inona no maha samy hafa ny faharisianao amin'ny izao fotoana izao sy io antony voalohany nanosika anao hanao AC io?

6. <u>Firotsahana an-tsitrapo sy valisoa ara-bola</u>

- Ny tambi-karama sy fidiram-bola
- Ny lanjan'ny firotsahana an-tsitrapo
- Ireo anton-javatra mety hampitombo ny fampaharetan'ny asa

Fanontaniana:

6.1. Araka ny valin'ny fanadiahadiana teo aloha dia latsaky ny 100 000 Ariary isam-bolana ny vola miditra ao an-tokatranon'ny AC amin'ny ankapobeny, misy fiantraikany amin'ny fanaovana asa an-tsitra-po ve izany?

6.2. Afaka lazainareo iza ve ireo rafitra mpandoa ny tambi-karaman'ny AC?

- Ny tondrozotran'ny governemanta dia milaza fa tokony hanao ny asany an-tsitrapo ny AC. Amin'ny fomba fijerinareo sy ny an'ny fiaraha-monina, inona ny maha zava-dehibe ny hoe atao an-tsitrapo io asa io?

6.3. Inona no hamaritanareo ny atao hoe asa an-tsitrapo?

6.4. Inona avy ireo sakana raha atao an-tsitrapo ny asan'ny AC?

6.5. Inona no fanovana tokony atao mikasika ny asa, ny tambin'asa, na ny tombotsoa hafa mba hampaharitra ny asa an-tsitrapo ataon'ny AC?

7. **Ahoana no hisafidiananareo ny asa ataonareo, hoe hidirako ity asa ity fa mampazoto**
8. **Inona avy no anton-javatra manosika anareo hazoto na tsia bebe kokoa amin'ny asa iray.**
9. **Azonareo zaraina aminay ve ireo andraikitry ny AC amin'ny ady amin'ny TB.**
10. **Inona ny fihatraikan'ny fifanarahana arak'asa amin'ny fahazotoanareo manao ny asan'ny AC momba ny areti-mifindra?**

Hadiahadio: Fanaovana ny asa an-tsitra-po, fisalasalana, fepetra

10.1. Hatraiza ny fahazotoan'ny na tsy fahazotoan'ny AC miasa momba ny areti-mifindra?

Hadihadio: fisian'ny ONG mpanohana, fiofanana, fanilikilihana, fifindran'ny aretina, fahasarotan'ny fanantonana ny marary.

10.2. Amin'ny maha mpiasan'ny fahasalamana anareo dia mety ho voairaka ianareo hiasa mikasika ny areti-mifindra, manao ahoana ny fihatraikan'izany amin'ny fahazotoanareo manao ny asanareo

11. Soso-kevitra hanohanana tsaratsara kokoa ny AC
   - Soso-kevitra azo ampiharina
   - Tohana amin'ny lafiny rehetra eo amin'ny fiainana

Fanontaniana:

11.1. Raha tazomina ve izao zava misy amin'izao fotoana izao ve ny mitazona sy manatsara ny ezaka amin'ny asa io?

11.2. Alao sary an-tsaina hoe manana hery majika ianao mamela anao hanatanteraka hetaheta iray izay afaka hanatsarana ny zavamisy amin'ny asan'ny AC, dia inona no ataonao?

11.3. Araka ny hevitrao, inona ihany koa no mety hanatsarana ny asan'ny AC? Amin'ny lafin-javatra maro eo amin'ny fiainana? Inona no ataonao raha

ianao manokana? Inona no azon'ny fiaraha-monina atao? Inona ny azon'ny mpanao politique sy mpanapa-kevitra atao?

Hadihadio: fiofanana, tambi-karama, fankasitrahana, fitaovana manokana, sns

12. Firotsahana amin'ny asa mikasika ny ady amin'ny areti-mifindra

12.1. Misy anton-javatra manokana na mahatonga ny CHW hisalasala handray anjara amin'ny ady amin'ireo aretina ireo ve? (ohatra ny tsy fahampian'ny ONG, ny tsy fahampian'ny fiofanana, ny fanilikilihana, ny fifindran'ny aretina

12.2. Inona ireo soso-kevitra hampitombona ny fandraisan'anjaran'ny AC amin'ny ady amin'ny areti-mifindra?

13. Famaranana

Misy zavatra tianao hanampiana ve?

Mbola manana fanontanina hafa ve ianao?

Fampafantarana mikasika ny tohiny (fanagonana angon-drakitra, fanadihadiana, fampielezana)

Fomba ifandraisana raha mbola misy ilàna izany any aoriana

Fisaorana

Focus Group Discussion Interview Guide (English version)

1. Introduction

Questions:

- Introduction of the interviewer and the study
- Information on good scientific practices, including confidentiality
- Obtaining informed consent
- Collection of sociodemographic data (affiliated health organization, age, sex, region)

2. Recruitment of CHWs

- How does one become a CHW?
- Selection criteria
- Decision-makers

Questions:

2.1. In your opinion, what are the characteristics of a good CHW?

Probe: Typical profile, attitudes, and skills required to become a CHW.

2.2. How does the selection process to become a CHW take place? What are the selection criteria?

Probe:
Selection process: is it the same or different depending on the location or other factors? Who is involved in the selection of CHWs? What is your opinion of the selection criteria?

2.3. Who is the most influential person in the selection of CHWs?

Probe: Their opinion regarding the key decision-maker.

3. Working Environment

- Challenges encountered
- Available resources and facilities
- Commitment

Questions:

3.1. From your perspective as a CHW, how would you describe your working environment in general?

Probe: Work atmosphere (positive or difficult), workload, occupational safety, risks, relationships with superiors, supervisors, and colleagues.

3.2. In your opinion, what are the most important aspects? Why?

Probe: Intrinsic and personal motivations such as recognition and gratitude, or material motivations.

3.3. Who is your supervisor, and how would you characterize the current supervision system?

3.4. What suggestions do you have to improve the quality of your work?

4. CHWs' Perceptions and Motivation

How do CHWs perceive their role within the community and the health sector?

Questions:

4.1. How do you perceive your role as a CHW within the health system?

Probe: To what extent do they feel indispensable? What could happen if CHWs were absent?

4.2. How do you perceive your role as a CHW within your community?

Probe: In relation to people with difficult access to healthcare, those living far away, vulnerable or stigmatized populations.

4.3. We have learned that your voluntary engagement as a CHW is very important for the community you serve. Could you tell us more about this?

4.4. What personal satisfaction do you gain from the services provided to the community?

4.5. What was your initial motivation for becoming a CHW?

Probe: Ask participants to share stories about how they became CHWs.

4.6. How is your current motivation different from your initial motivation? What happened?

Probe: Material and non-material motivations.

5. Volunteering and Financial Compensation

- Incentives and income
- Importance of volunteerism
- Factors enhancing sustainability

Questions:

5.1. According to previous scientific investigations, the majority of CHW households earn less than 100,000 MGA per month. In your opinion, does this influence the feasibility of voluntary work?

5.2. Could you describe the different sources of incentives or rewards for community health workers?

Government guidelines state that community health workers should perform their work voluntarily. From your perspective and that of society, how important is it that this work is done on a voluntary basis?

5.3. How do you define volunteerism?

5.4. What constraints do you face in carrying out voluntary tasks?

5.5. How feasible is it to work on a voluntary basis?
5.6. What changes should be made to activities or benefits in order to support CHWs' voluntary work?
6 Engagement in Different Areas of Work

6.1. How do you choose the areas of work in which you actively engage?

6.2. What factors influence your engagement in different areas?

6.3. Could you share your knowledge about the roles of community health workers in TB response?
7. Professional Contracts and Engagement in Communicable Disease Work

- Volunteerism
- Hesitation
- Conditions

Questions:

7.1. To what extent is a CHW motivated or not motivated to carry out work related to communicable diseases?

Probe: Lack of NGO involvement, lack of training, stigma, disease transmission risks, accessibility issues for patients.

As actors in public health, you may be assigned tasks related to communicable diseases such as TB.

7.2. How does this affect your motivation in carrying out your work?
8. Recommendations to Better Support CHWs

- Practical suggestions
- Support in different areas

Questions:

8.1. In the current context, is it possible to maintain and improve efforts regarding the tasks assigned to CHWs? Why?

8.2. Imagine that you had magical powers allowing you to make one wish that could improve CHWs' performance in the long term. What would your wish be?

8.3. In your opinion, what could improve the situation of CHWs in effectively carrying out their tasks?

You may think about different areas of life:

- What could you personally do?
- What could the community do?
- What could policymakers and other decision-makers do?

Probe: Equipment, incentives, supervision, training, performance feedback, recognition, gratitude.

9. Engagement in Communicable Diseases (TB & HIV/AIDS)

9.1. Are there factors that limit or influence the engagement of CHWs in response to these diseases?

(e.g., lack of NGO support, insufficient training, stigma, risk of infection, limited access to affected populations)

9.2. What specific recommendations would you make to increase CHWs' engagement in these areas?

9.3. Any additional questions?
10. Conclusion

Questions:

10.1. Would you like to add anything?

10.2. Any further questions?

- Information about the next steps (data collection, data analysis, dissemination)
- Reminder of contact details and the possibility of reaching out later if questions arise
- Thank you remarks

Guide d’entretien – Discussion de groupe / Agents communautaires (French Version)
1. Introduction

Questions :

- Présentation de l’intervieweur et de l’étude
- Informations sur les bonnes pratiques scientifiques, y compris la confidentialité
- Obtention du consentement éclairé
- Collecte des données sociodémographiques (organisation de santé d’affiliation, âge, sexe, région)

2. Recrutement des AC (Agents Communautaires)

Comment devient-on AC ?
Critères de sélection
Décideurs

Questions :

2.1. Selon vous, quelles sont les caractéristiques d’un bon AC ?
Relance : profil typique, attitudes et compétences requises pour devenir AC.

2.2. Comment se déroule le processus de sélection pour devenir AC ? Quels sont les critères de sélection ?
Relance :
Le processus de sélection est-il le même ou différent selon les localités ou d’autres facteurs ?

Qui participe à la sélection des AC ?

Que pensez-vous des critères de sélection ?

2.3. Qui est la personne la plus influente dans la sélection des AC ?
Relance : leur opinion sur le principal décideur.

3. Environnement de travail

- Difficultés rencontrées
- Ressources et infrastructures disponibles

- Engagement

Questions :

3.1. Comment décririez-vous votre environnement de travail en général en tant qu'AC ?
Relance : ambiance de travail (positive ou difficile), charge de travail, sécurité au travail, risques, relations avec les supérieurs, superviseurs et collègues.

3.2. Selon vous, quels sont les aspects les plus importants ? Pourquoi ?
Relance : motivations intrinsèques et personnelles telles que la reconnaissance et la gratitude, ou motivations matérielles.

3.3. Qui est votre superviseur et comment caractérisez-vous le système actuel de supervision ?

3.4. Quelles suggestions avez-vous pour améliorer la qualité de votre travail ?

4. Perceptions et motivation des AC

Comment les AC perçoivent-ils leur rôle dans la communauté et dans le système de santé ?

Questions :

4.1. Comment percevez-vous votre rôle d'AC dans le système de santé ?
Relance : Dans quelle mesure se sentent-ils indispensables ? Que se passerait-il en l'absence des ASC ?

4.2. Comment percevez-vous votre rôle d'AC au sein de votre communauté ?
Relance : en lien avec les personnes ayant un accès difficile aux soins, vivant loin, populations vulnérables ou stigmatisées.

4.3. Nous avons appris que votre engagement bénévole en tant qu'AC est très important pour la communauté que vous servez. Pouvez-vous nous en dire plus ?

4.4. Quelle satisfaction personnelle tirez-vous des services rendus à la communauté ?

4.5. Quelle était votre motivation initiale pour devenir AC ?
Relance : raconter les histoires de parcours.

4.6. En quoi votre motivation actuelle est-elle différente de votre motivation initiale ? Que s'est-il passé ?
Relance : motivations matérielles et non matérielles.

5. Bénévolat et compensation financière

- Incitations et revenus
- Importance du bénévolat
- Facteurs de durabilité

Questions :

5.1. Selon des études scientifiques antérieures, la majorité des ménages d'AC gagnent moins de 100 000 MGA par mois. Pensez-vous que cela influence la faisabilité du travail bénévole ?

5.2. Pouvez-vous décrire les différentes sources d'incitations ou de récompenses pour les agents de santé communautaires ?
Les directives gouvernementales indiquent que les AC doivent travailler de manière bénévole. Selon vous et la société, quelle est l'importance de ce bénévolat ?

5.3. Comment définissez-vous le bénévolat ?

5.4. Quelles contraintes rencontrez-vous dans l'exécution des tâches bénévoles ?

5.5. Dans quelle mesure est-il faisable de travailler sur une base bénévole ?

5.6. Quels changements devraient être apportés aux activités ou aux avantages pour soutenir le travail bénévole des AC ?

6. Engagement dans différents domaines d'activité

6.1. Comment choisissez-vous les domaines dans lesquels vous vous engagez activement ?

6.2. Quels facteurs influencent votre engagement dans différents domaines ?

6.3. Pouvez-vous partager vos connaissances sur le rôle des AC dans la réponse à la tuberculose ?

7. Contrats professionnels et engagement dans les maladies transmissibles

- Bénévolat
- Hésitation
- Conditions

Questions :

7.1. Dans quelle mesure un AC est-il motivé ou non à réaliser des activités liées aux maladies transmissibles ?
Relance : manque d'implication des ONG, manque de formation, stigmatisation, risques de transmission, difficultés d'accès des patients.
En tant qu'acteurs de santé publique, vous pouvez être amenés à effectuer des tâches liées à des maladies transmissibles comme la tuberculose.

7.2. Comment cela influence-t-il votre motivation dans l'exécution de votre travail ?

8. Recommandations pour mieux soutenir les AC

- Suggestions pratiques
- Soutien dans différents domaines

Questions :

8.1. Dans le contexte actuel, est-il possible de maintenir et d'améliorer les efforts concernant les tâches confiées aux AC ? Pourquoi ?

8.2. Imaginez que vous avez des pouvoirs magiques vous permettant de faire un vœu pour améliorer durablement la performance des AC. Quel serait ce vœu ?

8.3. Selon vous, qu'est-ce qui pourrait améliorer la situation des AC dans l'accomplissement efficace de leurs tâches ?
Vous pouvez réfléchir à différents niveaux :

- Que pouvez-vous faire personnellement ?
- Que peut faire la communauté ?
- Que peuvent faire les décideurs politiques et autres acteurs ?
  Relance : équipements, incitations, supervision, formation, retour de performance, reconnaissance, gratitude.

9. Engagement dans les maladies transmissibles (TB & VIH/SIDA)

9.1. Existe-t-il des facteurs qui limitent ou influencent l'engagement des AC dans la réponse à ces maladies ?
(ex. : manque de soutien des ONG, formation insuffisante, stigmatisation, risque d'infection, accès limité aux populations concernées)

9.2. Quelles recommandations spécifiques feriez-vous pour augmenter l'engagement des AC dans ces domaines ?

9.3. Autres questions supplémentaires ?
10. Conclusion

Questions :

10.1. Souhaitez-vous ajouter quelque chose ?

10.2. Avez-vous d’autres questions ?

- Informations sur les prochaines étapes (collecte, analyse, diffusion des données)
- Rappel des contacts et possibilité de recontacter ultérieurement en cas de questions
- Remerciements
