# Supplementary material for: Voices from the front line: Community health workers’ perspectives on effectiveness and needs in Madagascar’s tuberculosis response
Source: PLOS Glob Public Health. 2026 Oct 22;6(10):e0005797. doi: 10.1371/journal.pgph.0005797 (PMC13690241; doi:10.1371/journal.pgph.0005797)
Supplement: Supplementary file 2 [file pgph.0005797.s002.pdf]

COREQ (Consolidated criteria for Reporting Qualitative research) Checklist

topic	Item No	Information reported in the manuscript	Reported on
**Domain 1: Research team and reflexivity**			
Interviewer/facilitator	1	Focus group discussions were conducted by a trained qualitative research team, including a coordinator, a lead moderator, and a note taker.	Methods, data collection
Credentials	2	Not explicitly reported (academic or professional qualifications of moderators not detailed).	Methods, data collection
Occupation	3	Moderators were external to the communities and not affiliated with local health facilities or community programmes.	Methods, data collection
Gender	4	2 male and 1 female.	Methods, data collection
Experience and training	5	Moderators were trained in qualitative research; the discussion guide was pilot tested.	Methods, data collection
Relationship with participants			
Relationship established	6	No prior relationship between data collectors and participants.	Methods, participant recruitment and data collection
Participant knowledge of the interviewer	7	Participants were informed that the study aimed to generate evidence to strengthen community health programmes.	Methods
Interviewer characteristics	8	Implicitly addressed: external data collectors were used to reduce social desirability bias; constructivist–interpretivist orientation stated.	Methods, data collection
**Domain 2: Study design**			
Theoretical framework			
Methodological orientation and Theory	9	Inductive reflexive thematic analysis within a constructivist–interpretivist paradigm (Braun & Clarke).	Methods, study design
Participant selection			
sampling	10	Two-stage purposive sampling (selection of CSBs, then CHWs).	Methods, data collection
Method of approach	11	CHWs were invited via selected CSBs, with logistical support from a local partner (no role in data collection or analysis).	Methods, data collection
Sample size	12	26 community health workers.	Methods collection, table 1
Non-participation	13	Not reported (no refusals or dropouts).	N/A
Setting			

Setting of data collection	14	FGDs conducted in community-accessible locations (implicit).	Methods, data collection
Presence of non-participant	15	None (only participants and research team present).	Methods, data collection
Description of sample	16	Detailed (gender, age range, role, area, region – Table 1).	Results
Data collection			
Interview guide	17	Semi-structured thematic guide, pilot tested.	Method, data collection
Audio/visual recording	18	Audio-recorded with participant consent.	Method, data collection
field notes	19	Yes, a note taker was present during FGDs.	Method, focus group discussions
Duration	20	Mean duration 88 minutes.	Method, focus group discussion
Data saturation	21	Thematic sufficiency reached by the fifth FGD (no new codes emerged).	Not reported
Participant checking	22	Not conducted (not reported).	Methods
**Domain 3: analysis and findings**			
Data analysis			
Number of data coders	23	One primary analyst, with review and discussion of selected excerpts by a second researcher.	Methods, data analysis
Description of coding tree	24	a dynamic, iterative codebook was developed and refined during analysis.	Method, data analysis
Derivation of themes	25	Inductive, data-driven.	Method, data analysis
software	26	NVivo (QSR International).	Method, data analysis
Participant checking	27	Not conducted (not reported).	NA
Reporting			
Quotations presented	28	Extensive verbatim quotations included.	Results
Quotations identified	29	Quotations labelled by gender, role, and region.	Results
Data and findings consistent	30	Strong alignment between data and themes.	Results
Clarity of major themes	31	Five clearly defined interlinked themes.	Results
Clarity of minor themes	32	Sub-dimensions clearly described within themes.	Results
