# Supplementary material for: Voices from the front line: Community health workers’ perspectives on effectiveness and needs in Madagascar’s tuberculosis response
Source: PLOS Glob Public Health. 2026 Oct 22;6(10):e0005797. doi: 10.1371/journal.pgph.0005797 (PMC13690241; doi:10.1371/journal.pgph.0005797)
Supplement: Supplementary file 3 [file pgph.0005797.s004.pdf]

Response to reviewers' and editor's comments

Manuscript title: Voices from the front line: community health workers' perspectives on effectiveness and needs in Madagascar's tuberculosis response

Manuscript ID: PGPH-D-25-03991

Date: February 23rd, 2026

Dear reviewers, dear editor,

We would like to thank the reviewers and editor for their helpful and constructive feedback. Please see below a point-by-point response to the comments from each reviewer and the editor.

We are hopeful that these changes meet your expectations. We look forward to your feedback.

Yours faithfully,

The authors

Journal Requirement	
1_Please ensure that your Ethics Statement is available in its entirety at the beginning of your Methods section, under a subheading 'Ethics Statement'.	Thank you for this reminder. We have revised the manuscript and shifted the full Ethics Statement to the beginning of the Methods section, in accordance with the journal requirements **(now lines 96-105).**
2_We have noticed that you have uploaded Supporting Information files, but you have not included a list of legends. Please add a full list of legends for your Supporting Information files after the references list.	Thank you for bringing this to our attention. We now include the list of legends for the Supporting Information file at the end of the manuscript after the references:<br><br>***Supporting information (lines 714-716):***<br>● **S1 Checklist.** Completed Consolidated Criteria for Reporting Qualitative Research (COREQ) checklist.
3_All PLOS journals now require all data underlying the findings described in their manuscript to be freely available to other researchers, either<br>1. In a public repository,<br>2. Within the manuscript itself, or<br>3. Uploaded as supplementary information.<br><br>This policy applies to all data except where public deposition would breach compliance with the protocol approved by your research ethics board. If your data cannot be made publicly available for ethical or legal reasons (e.g., public availability would compromise patient privacy), please explain your reasons by return email and your exemption request will be escalated to the editor for approval. Your exemption request will be handled independently and will not hold up the peer review process, but will need to be resolved should your manuscript be accepted for publication. One of the Editorial team will then be in touch if there are any issues.<br><br>If the reviewer comments include a recommendation to cite specific previously published works, please review and evaluate these publications to determine whether they are relevant and should be cited. There is no requirement to cite these works unless the editor has indicated otherwise.	Thank you for outlining the journal's data availability policy. The qualitative data underlying this study consist of full FGD transcripts containing detailed contextual information about participants' roles, regions, and working conditions. According to the approved protocol and informed consent procedures (CEERINSPC/IRB; Decision No. 003/2023), participants were informed that their data would be shared only among the named partner institutions and not with third parties. Public deposition of full transcripts in an open repository would therefore be inconsistent with the approved data protection framework. In addition, given the limited number of participants and the specificity of contexts, even fully de-identified transcripts would carry a risk of deductive disclosure.<br><br>We have formally requested an exemption from public data deposition by return email and are happy to provide any further documentation required. De-identified excerpts to support the study findings are included within the manuscript. We revised our Data availability statement accordingly as follows:<br><br>***Data availability (lines 570-575):***<br>"The data supporting this study are not publicly available due to confidentiality considerations and the risk of deductive disclosure. De-identified excerpts are included in the manuscript. Fully anonymised data may be made available upon reasonable request to the corresponding author, subject to approval by the relevant ethics committee and the establishment of a data-sharing agreement to protect participant confidentiality."
**Comments by Reviewer 1**	
1) Introduction<br>I lack a clearly stated research aim or question. The closest I found was what was mentioned<br>Line 87-88 that the "research initiative aimed at strengthening Madagascar's community health workforce". This is furthermore quite a broad and not very specific aim. I would thus suggest for the authors to more clearly state the papers´ aim.	Thank you for this important feedback. We have revised both the abstract and the background section to clearly and explicitly state the study's aim. The revised text is provided below.<br><br>***Abstract (lines 25-27):***<br>"This study aims to examine the lived experiences of Malagasy CHWs engaged in TB programme activities and to develop context-specific recommendations to strengthen their effectiveness in Madagascar's TB response."<br><br>***Background (lines 81-93):***<br>"Despite their central role in delivering essential health services in Madagascar, limited evidence captures CHWs' own perspectives on the challenges they face

	and the support they require to work effectively within the health system. To address this gap, this study aims to examine the lived experiences and perceived constraints of Malagasy CHWs involved in TB programme support, and to identify context-specific recommendations for improving CHW effectiveness in the TB response. We use TB programme support as a tracer condition for UHC, drawing on CHWs' perspectives to analyse systemic barriers and opportunities for improvement. This study is part of a broader intersectoral action research initiative aimed at strengthening Madagascar's community health workforce [15]."
2) Method<br><br>a) The study design and study setting is well detailed.<br>Line 121- 127. I get a sense of the inclusion criteria, where there are also exclusion criteria. Maybe state these more explicitly.	Thank you for this helpful suggestion. We have revised the Methods section to explicitly state the inclusion and exclusion criteria for participant selection. We added the following text:<br><br>***Sampling and selection of participants (lines 148-150):***<br>"CHWs were excluded if they had less than 12 months of experience in their role, were not officially registered as CHWs at the selected health centers, or declined to provide informed consent."
b) Line 140-143. How, where and with whom were the thematic guide pilot-tested?	Thank you for this clarification request. We have revised the text to specify how, where, and with whom the thematic guide was pilot-tested, and to describe adjustments made after the pilot. The addition reads as follows:<br><br>***Data collection (lines 167-170):***<br>"The guide was pilot-tested through two mock interviews with CHWs from outside the study area to assess clarity, relevance, flow, and duration. These participants were not included in the final study sample. Minor revisions to question wording and sequencing were made based on the feedback."
c) Line 147. How was written and oral informed consent gathered? Where participants informed about the aims of the study, the data collection and analysis process and potential benefits of the study?	Thank you for highlighting the need for clarification. We have revised the text accordingly and added further details on the procedure. The added information reads as follows:<br><br>***Data collection (lines 174-179):***<br>"Prior to each FGD, the facilitator read aloud an information sheet detailing the study objectives, data collection and analysis procedures, and potential risks and benefits. Participants were informed about the voluntary nature of participation, their right to withdraw at any time, and the measures taken to ensure confidentiality. They were given the opportunity to ask questions before providing written informed consent. Oral consent was reconfirmed at the start of each FGD."
d) Line 152. Please motivate the choice of analysis approach and I would suggest adding some references to support the choice of analysis.	Thank you for this important suggestion. We have now clarified the rationale for our choice of analytic approach and added references to foundational methodological papers by Braun and Clarke to support this choice and to clarify the epistemological alignment of our analysis. The section now reads as follows:<br><br>***Data analysis (lines 185-199):***<br>"We used the French transcripts alongside Malagasy recordings to analyze the data through reflexive thematic analysis (TA) as outlined by Braun and Clarke [22,23]. The analysis approach was selected for three main reasons: first, this study aims to understand CHWs experiences as diverse, complex, and subjective while recognizing that the researchers' positionality impacts the analysis and findings. It is situated in a constructivist-interpretivist paradigm. Braun and Clarke's reflexive TA centers this process of developing understanding between the data and the researchers, drawing attention to

	reflexivity as a core element of analysis. Second, reflexive TA suits the study's inductive and reiterative approach to analysis, which was selected because it allows themes to be developed directly from participants' accounts rather than being constrained by a predefined theoretical framework. No prior theory guided the analysis; this was an intentional methodological choice consistent with the exploratory and<br>formative nature of the study, allowing unexpected insights to emerge while remaining grounded in participants' perspectives. Third, our goal was to not merely uncover descriptive but also latent meaning, particularly in the development of recommendation based on the data."
e) Line 154-160 Perhaps a table or illustration would help the reader in grasping the analysis process.	Thank you for this helpful suggestion. We agree that a visualization of the analytical process improves clarity and transparency. We added a Figure 1 to the manuscript illustrating the iterative and reflexive phases of the analysis process:<br><br>***Data analysis (lines 208-209):***<br><br>**Figure 1.** Iterative reflexive thematic analysis process used to examine community health workers' perspectives.<br>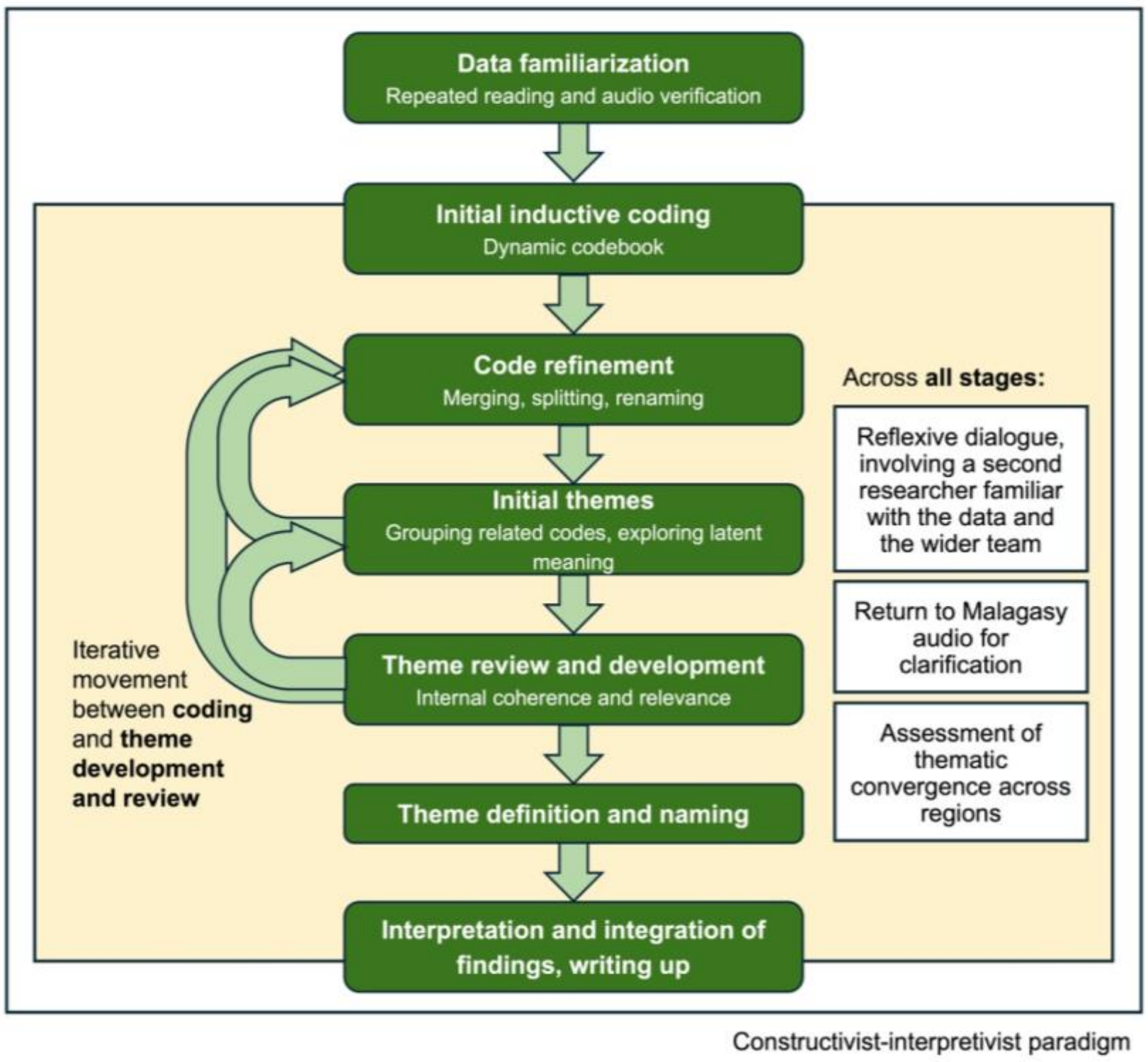

f) Line 161- 165 Clarify the motivation for focusing the analysis with the first author and only having a second author coming in to review specific excerpts and codes.	Thank you for this request for clarification. In line with reflexive thematic analysis (Braun & Clarke), coding and theme development were conceptualized as interpretive, researcher-led processes rather than consensus-based or inter-coder reliability procedures. The first author therefore conducted the primary coding to ensure depth of engagement and analytic coherence across the dataset. A second researcher was involved to enhance reflexivity rather than to establish inter-coder agreement. We have revised the section to more clearly explain our rationale in the manuscript:<br><br>***Data analysis (lines 216-221):***<br>"In line with Braun and Clarke's arguments, the purpose of integrating a second researcher was related to the practice of reflexivity. The research team came to this project with different degrees of distance to the reality of CHWs, depending on their background and experiences. We leveraged these positionalities by approaching the data together and discussing sections that were unclear, had multiple meanings, or were particularly crucial to the research questions."
g) Line 176-177 As mentioned regarding line 147 clarify the process through which this oral and written consent was obtained.	Thank you for this important point. We have revised the manuscript to clarify the informed consent procedure, explaining that prior to each FGD, the facilitator distributed the consent form, read it aloud, and allowed time for questions. Participants who agreed to take part provided written consent before the discussion began. The corresponding additions are detailed in the **response to comment 2) c).**
3) Results<br><br>The results are overall well detailed with excerpts of participants discussions and connecting narrative highlighting the key themes. However, recurring sentences seem to go further into the authors reflective analysis which makes it a bit more difficult what is the results of the discussions and originates from the participants discussions and what is the authors interpretations. I have included a number of examples of this below.<br><br>a) Line 203-206, This has parts that are more analytical reflection and perhaps would be better suited for the discussion section, leaving the results section more focused on reporting. Its a good reflection just unsure its placement is right.	Thank you for this helpful observation. We agree that clearer separation between participants' accounts and broader analytical reflection improves readability. We have revised the Results section to ensure that it focuses more explicitly on reporting participants' perspectives and have moved interpretive reflections to the Discussion section.<br><br>Regarding comment 3) a), we shifted the content to the discussion section.
b) Line 218-219 This sentence also seem to more belong in the discussion.<br>c) Line 221-223 This is another sentence that could work better in the discussion.<br>d) Line 246-248, Consider moving to discussion.	Thank you; we have revised these sentences to ensure the results section focuses more clearly on participants' reported accounts, removed interpretive framing and integrated the revised content into the Discussion.

e)	Line 250- onward. This theme is much better in focusing on reporting on what the participants said and discussed without adding further analytical framing that more belongs to the discussion.	Thank you for the helpful observations and feedback regarding the balance between results reporting and interpretation. We improved the consistency throughout the results section.
f)	Line 286-287. I suggest to be clear in the wording, likely participants expressed perceptions as part of the FGDs.	Thank you for this helpful clarification. We have revised these lines to more clearly reflect that these statements represent participants' expressed perceptions during the FGDs. The text now reads as follows:<br><br>***Formative and collaborative supervision (lines 325-328):***<br>"CHWs reported perceiving the current supervision model as top-down or punitive and disconnected from the realities of their work. They expressed the desire for a shift from a control-based approaches to more formative, supportive, and collaborative supervision focused on guidance rather than fault-finding or punishment:"
g)	Line 324-325. Clarify if this is what was expressed by the participants or the authors analysis.	Thank you; we have removed this interpretation from the results section and integrated the revised content into the Discussion.
h)	Line 328-330. Please be clear if this is what was expressed by the participants.	Thank you! We have clarified that this is a direct quotation from a participant as follows (lines 370-373):<br><br>***Access to work tools, equipment, and transport (lines 365-368):***<br>"During FGDs, a frequently cited obstacle was disrupted availability of essential tools and resources for carrying out duties. Participants emphasized that these prerequisites were viewed as needing systematic and permanent rather than exceptional provision. One female CHW explained:"
i)	Line 341-342. Maybe be clear that we only know that its what CHWs expressed rather than what they did.	We thank the reviewer for this comment. We have clarified that this statement reflects CHWs' perceptions expressed during FGDs as follows:<br><br>***Basic functional equipment (lines 378-381):***<br>"Participants reported that the absence of basic resources such as forms, registers, and health education materials (e.g., posters, brochures) prompted them to improvise or rely on personal resources, which they perceived as affecting quality, consistency, and credibility of their work. One female explained:"
j)	Line 349-351. I would suggest formulating it as "CHWs expressed how they often…"	Thank you! We have revised accordingly:<br><br>***Tools to improve feasibility and efficiency (lines 386-390):***<br>"CHWs described walking for hours to reach patients or health centers, which they experienced as physically demanding and financially costly. Without access to bicycles or travel allowances (called "solom-paladia" in Malagasy, meaning walking allowance), CHWs explained that they often had to cover transportation costs out of pocket. Some reported that when they could not afford transport, visits and activities were postponed or missed."
k)	Line 368. Clarify that this is what was expressed.	Thank you! We have revised accordingly:<br><br>***Tools to improve feasibility and efficiency (lines 409-410):***<br>In contrast, they explained that lacking tools diminished their sense of professionalism and authority in the eyes of the community.

l) Line 398. Maybe clarify that it is what was expressed by the participants.	Thank you! We have revised accordingly:<br><br>***Recognition and integration into the health system (lines 438-439):***<br>"Many CHWs expressed that institutional recognition would strengthen their credibility, clarify roles, and foster better collaboration with professional healthcare staff."
4) Discussion<br><br>a) The discussion is overall well-presented and raises multiple important points and contributions of the paper, as exemplified in Line 418-423 where analytical reflection is very relevant. However, I have some minor comments.<br>Line 410-411. Maybe reference what research has been done on CHW´s in Madagascar if any.<br>While UHC was outlined as the key focus with TB as a tracer condition programme support its only directly mention and engaged with on two points in the discussion, line 409 and 495. I would suggest that the authors more actively engage with UHC in the discussion	Thank you!<br>1) We agree that the discussion should more explicitly situate our findings within the existing evidence base on CHWs in Madagascar and also now more actively engage with UHC as follows:<br><br>***Discussion (lines 449-455):***<br>"From a UHC perspective, adequately supported and well-governed CHW programs are essential for equitable service delivery. Despite persistently poor health indicators, minimal progress toward UHC [11], and the presence of a large CHW workforce in Madagascar, there is little peer-reviewed evidence on CHWs' working conditions and the support they actually receive. While prior research has documented workload pressures and socioeconomic constraints [24], less attention has been given to how CHWs themselves experience the structural conditions shaping their work."<br><br>2) We also clarify how using TB as a tracer illuminates system bottlenecks that affect UHC progress more broadly:<br><br>***Discussion (lines 456-465):***<br>"We used TB as a tracer condition to examine how these structural factors operate in a demanding service area. Engagement in TB care requires sustained follow-up, infection prevention, and stigma-sensitive communication, and is therefore particularly sensitive to weaknesses in training, supervision, and material support [25]. Our findings show that infection risk, stigma, and the need for sustained follow-up were compounded by insufficient training, transport shortages, and inconsistent material support. Although some challenges, such as limited access to personal protective equipment, are specific to infectious disease contexts, most priorities identified by CHWs extend beyond TB programming and point to broader system-level governance weaknesses affecting CHW effectiveness."
5) Data availability<br>Please refer to the journal's requirements of data availability.	Thank you for the comment regarding data availability. We have revised the Statement to align with the journals' requirements but also towards ensuring ethical safeguard as requested by the local Malagasy Ethics Committee. We amended the Data Availability Statement as follows:<br><br>***Data availability (lines 570-575):***<br>"The data supporting this study are not publicly available due to confidentiality considerations and the risk of deductive disclosure. De-identified excerpts are included in the manuscript. Fully anonymised data may be made available upon reasonable request to the corresponding author, subject to approval by the relevant ethics committee and the establishment of a data-sharing agreement to protect participant confidentiality."<br><br>We have formally requested an exemption from public data deposition from the Academic Editor (cf. **response to Journal Requirements 3_**).
Comments by Reviewer 2	
This manuscript addresses an important topic regarding community health workers	Thank you for your careful review and for highlighting your concerns; we appreciate the opportunity to further strengthen our manuscript. In response,

and TB care in Madagascar and is clearly written with strong policy framing. However, significant methodological and analytical weaknesses limit its suitability for publication in its current form.	we have substantially revised the Methods and Discussion sections to improve methodological transparency and analytical depth. Please find our **detailed responses and corresponding changes and additions below.** We have also addressed Reviewer 1's suggestions regarding methodological and analytical clarification, as **outlined above**. We hope that these revisions adequately address the concerns raised and enhance the overall rigor and clarity of the manuscript.
1) Insufficient Qualitative Rigor<br><br>a) The manuscript does not adequately demonstrate data saturation, coding procedures, reflexivity, or analytical validation. The methodological transparency expected for qualitative research is lacking.	Thank you for raising these important concerns regarding qualitative rigor. We have revised the manuscript to improve methodological transparency as follows:<br>● Data sufficiency: Our analysis followed a reflexive thematic approach, which does not conceptualise "data saturation" in positivist terms (Braun & Clarke, 2021). For clarity, we have now explained how we assessed the adequacy of our dataset. Drawing on the concept of "information power" (Malterud et al., 2015), we explain why the focused research aim, specificity of the participant group, and depth of discussion supported sufficient analytical depth. We have added this clarification to the Methods section as follows:<br><br>***Data analysis (lines 223-229):***<br>"Given the focused research aim and the specificity of the participant group, we assessed data adequacy in terms of depth, richness, and thematic convergence rather than numerical saturation. Across the five FGDs, participants consistently articulated similar core priorities and challenges, while also providing detailed contextual examples. The recurrence of key themes across two contrasting regions supported the sufficiency of the dataset for addressing the study objectives."<br><br>● Coding procedures: We expanded the description of the coding and theme development process and added a figure more clearly illustrating the analytical steps, as also suggested by Reviewer 1. We would therefore like to refer to our **responses to Reviewer 1 comments 2) d), e), and f).**<br><br>● Reflexivity: We now include a **reflexivity and positionality statement (lines 751-761)**. In addition, we clarified in the Data analysis section how reflexive discussions between authors were used to review ambiguous excerpts and reflect on coding decisions:<br><br>***Data analysis (lines 214-219):***<br>"A second researcher was also involved to review specific excerpts and discuss the relevance or formulation of certain codes. In line with Braun and Clarke's arguments, the purpose of integrating a second researcher was related to the practice of reflexivity. The research team came to this project with different degrees of distance to the reality of CHWs, depending on their background and experiences. We leveraged these positionalities by approaching the data together and discussing sections that were unclear, had multiple meanings, or were particularly crucial to the research questions."<br><br>● Analytical validation: We clarified how interpretative decisions were discussed within the research team, how ambiguous excerpts were revisited in the original-language transcripts and audio recordings, and how candidate themes were refined based on internal coherence, relevance to the research question, and grounding in participants' narratives. For the corresponding changes, we kindly refer you to the changes provided regarding the **response on Reflexivity** (***Data***

	analysis (lines 214-219)) and in ***Figure 1*** **(response to Reviewer 1 comment 2) e)).**<br><br>We believe these revisions substantially strengthen the transparency and rigor of the manuscript.
b) Descriptive Rather Than Analytical Findings<br>The themes presented (training, supervision, payment, tools, recognition) are well-known in CHW literature and are described rather than deeply analyzed.	Thank you for this important comment. We agree that the thematic domains identified are already discussed in the CHW literature. Our intention was not to claim novelty at the level of thematic categories but to analyze how these domains are experienced and interpreted by CHWs within the Malagasy setting - a highly fragmented, partner-driven implementation context. In the revised manuscript, we strengthened the analytical framing as follows:<br><br>1. We clarified that the central analytical contribution lies not in listing support needs, but in highlighting the implementation gap between formal policy commitments and frontline realities. The Discussion now more explicitly examines how reliability, predictability, and fulfilment of entitlements emerge as governance concerns rather than operational deficits; we emphasize the "how" CHWs are supported rather than the "what" CHWs need **(Discussion lines 466-481).**<br><br>2. We also tried to deepen the analysis of fragmentation by showing how uneven remuneration, inconsistent training access, and punitive supervision are experienced by CHWs as injustice and demotivation. With this we aim to connect macro-level programme architecture to relational and motivational consequences for CHWs **(Discussion lines 488-493).**<br><br>3. We sharpened the argument that the core tension identified by CHWs is less volunteer status per se than the absence of predictable protections **(Discussion lines 510-525).**<br><br>4. Throughout the discussion section, we reduced descriptive repetition and made interpretive steps more explicit.<br><br>We hope the revised manuscript now more clearly demonstrates the analytical depth of the study while remaining grounded in CHWs' narratives.
c) TB Not Effectively Used as a Tracer Condition<br>Although TB is introduced as a tracer for UHC, the findings are not TB-specific and do not analytically leverage this framing.	Thank you for this thoughtful observation. We agree that the tracer logic has not been sufficiently clear and analytically insufficiently visible in the manuscript. Our intention in using TB as a tracer condition was not to produce TB-specific clinical findings, but to "stress-test" the community health system through a demanding service area that requires sustained follow-up, infection prevention, stigma-sensitive communication, and reliable referral pathways. We selected TB because it makes structural weaknesses particularly visible.<br>In the revised manuscript, we have clarified this rationale more explicitly in both the Introduction and the Discussion section. Specifically:<br><br>1. We now more clearly explain that TB was used to examine how broader governance and support structures operate under conditions of heightened complexity (e.g., infection risk, stigma, long-term adherence monitoring) when introducing the study aim and approach:<br><br>***Introduction (lines 85-91):***<br>"TB program support was used as a tracer condition for UHC because engagement in TB places particular demands on CHWs, including sustained patient follow-up, infection control, stigma-sensitive communication, and

<table>
<tr><td></td><td>reliable referral pathways. These features intensify the need for consistent support and effective governance, thereby exposing gaps that may be less visible in less stigmatized or more episodic areas of care. Drawing on CHWs' perspectives, we analyze systemic barriers and opportunities for improvement."

Discussion (lines 456-461):
"We used TB as a tracer condition to examine how these structural factors operate in a demanding service area. Engagement in TB care requires sustained follow-up, infection prevention, and stigma-sensitive communication, and is therefore particularly sensitive to weaknesses in training, supervision, and material support [25]. Our findings show that infection risk, stigma, and the need for sustained follow-up were compounded by insufficient training, transport shortages, and inconsistent material support."

2. We strengthened the analytical link between TB-specific demands and the system-level weaknesses described by CHWs and show that many of the priorities identified extend beyond TB programming:

Discussion (lines 461-465):
"Although some challenges, such as limited access to personal protective equipment, are specific to infectious disease contexts, most priorities identified by CHWs extend beyond TB programming and point to broader system-level governance weaknesses affecting CHW effectiveness."

We appreciate this comment, as it effectively helped us sharpen the conceptual clarity of the tracer approach and make its contribution more analytically explicit.</td></tr>
<tr><td>d) Discussion Overreaches the Data Governance and policy critiques in the discussion are not sufficiently grounded in the empirical findings</td><td>Thank you for this important comment. We appreciate the concern that elements of the Discussion may have appeared to extend beyond the empirical findings. We thoughtfully revised the manuscript. In the revised version, we have carefully restructured and tightened the Discussion to ensure that all governance- and policy-related reflections are explicitly grounded in the empirical data presented in the Results. The following specific changes have been made:
1. We now anchor the discussion around (1) fragmentation, (2) inequity, and (3) unpredictability directly and explicitly in participants' accounts, such as those of uneven remuneration, inconsistent training access, fragmented supervision, and unfulfilled commitments.
2. We reformulated and partially reduced normative statements that could be interpreted as independent critique.
3. We clarified when arguments represent interpretive implications derived from CHWs' testimonies rather than empirical claims extending beyond the dataset.
4. We strengthened transitions between Results and Discussion to make the analytical steps more transparent and proportionate to the qualitative evidence.

Our intention is to situate CHWs' lived experiences within the broader governance environment shaping them, not to advance claims beyond the scope of the data. We hope that the revised version now more clearly signals this interpretive move and remains closely aligned with participants' narratives.</td></tr>
<tr><td>e) Data Availability Does Not Meet PLOS Standards</td><td>Thank you for the comment regarding data availability. We have revised the Statement to align with the journals' requirements but also towards ensuring</td></tr>
</table>

Available upon request" is insufficient and non-compliant with PLOS data-sharing policy.	ethical safeguard as requested by the local Malagasy Ethics Committee. We amended the Data Availability Statement as follows:<br><br>***Data availability (lines 570-575):***<br>"The data supporting this study are not publicly available due to confidentiality considerations and the risk of deductive disclosure. De-identified excerpts are included in the manuscript. Fully anonymised data may be made available upon reasonable request to the corresponding author, subject to approval by the relevant ethics committee and the establishment of a data-sharing agreement to protect participant confidentiality."<br><br>We have formally requested an exemption from public data deposition from the Academic Editor (cf. **response to Journal Requirements 3_**).
f) Limited Novel Contribution<br>The study offers contextual insight but little new conceptual or theoretical contribution.	Thank you for your comment. We agree that much of the global literature has examined challenges faced by CHWs in various contexts. However, we believe this study contributes in the following ways:<br>1. Our study provides the first in-depth qualitative analysis of CHWs' perspectives within Madagascar's TB response, addressing a documented evidence gap in this setting.<br>2. By using TB programme support as a tracer condition for UHC, the study advances understanding of how disease-specific programmes intersect with broader health system constraints at the community level.<br>3. Our findings contribute to ongoing debates on the professionalization and supervision of CHWs by highlighting how supervisory models are experienced and interpreted by CHWs themselves in a low-resource setting.<br>We have revised the Discussion sections throughout to more explicitly articulate these conceptual contributions and to situate our findings within broader theoretical and policy debates.
