# Supplementary material for: Voices from the front line: Community health workers’ perspectives on effectiveness and needs in Madagascar’s tuberculosis response
Source: PLOS Glob Public Health. 2026 Oct 22;6(10):e0005797. doi: 10.1371/journal.pgph.0005797 (PMC13690241; doi:10.1371/journal.pgph.0005797)
Supplement: Supplementary file 4 [file pgph.0005797.s005.docx]

**Response to comments**

**Manuscript title:** Voices from the front line: community health workers’ perspectives on effectiveness and needs in Madagascar’s tuberculosis response

**Manuscript ID:** PGPH-D-25-03991

**Date:** April 17^th^, 2026

Dear reviewers, dear editor,

We thank the reviewers and editor for their helpful feedback and engagement with our manuscript in this second round. Please find below our point-by-point responses to the comments raised. We hope that these changes address the remaining concerns. We look forward to your further consideration.

Yours faithfully,

The authors

| Comments by reviewer 3 | |
| --- | --- |
| Please ensure that you include your reflexivity statement - it does not appear to be in either the clean or tracked version at the lines you listed. | Thank you for flagging this; we apologise for any confusion by stating incorrect line numbers. The **Reflexivity and Positionality statement** is present in both the clean and tracked versions of the manuscript, under the heading "Reflexivity and positionality", at **lines 568–578**:  *“****Reflexivity and positionality***  *The two main authors brought different perspectives to this study. OR is a Malagasy social scientist working in community health at the NGO Doctors for Madagascar. Her familiarity with the local context and health system supported nuanced interpretation of participants’ accounts. NM is a clinician and global health researcher based in Europe and formerly served as a board member of Doctors for Madagascar. She is a research group lead at Charité – Universitätsmedizin Berlin. Her position allowed for critical distance in interpreting the findings. The two authors are aware that their social and institutional roles could influence data interpretation. Throughout the analysis, they held regular discussions to reflect on assumptions, question interpretations, and consider how their positions shaped the analytic process.”* |
| Comments by reviewer 4 |  |
| Comment 1:  I note that the included respondents are female dominated in one site, and male dominated in the other. Please include in your methodology description the extent to which this reflects the overall situation of active CHWs in the two sites. Also discuss in your limitations the possible influence of this to the study results. | Thank you for this insightful observation. We have clarified this point in the **Methods** section (**lines 150–157**) as follows:  *“Recruitment and discussion-group composition aimed to capture a wealth of perspectives and field realities by balancing tenure, role, and catchment profile, as well as gender where the available workforce allowed. The observed gender distribution reflects the composition of the active CHW workforce at the selected health centers in each region, as confirmed with local health facility staff prior to recruitment.”*  We have also added a sentence to the limitations paragraph in the **Discussion** acknowledging the potential influence of this distribution on the findings (**lines 550–553**):  *"Additionally, interpretation should consider that the gender composition of participants reflects contextual workforce structures in urban and rural settings and may have influenced the perspectives expressed."* |
| Comment 2:  It may be useful to include a sex-based approach in analysis of the results, to reflect any differences in views expressed by females and males. | We appreciate this suggestion and agree that a sex-based analysis would provide valuable additional insights. However, the primary objective of this study was to examine CHWs' shared experiences and priorities rather than to compare subgroups. Furthermore, the number of participants within each sex group was limited, which would not allow for a robust or meaningful comparative analysis. We fully agree and note that future research specifically designed to examine sex-disaggregated perspectives would be well placed to address this important question. |
