# Supplementary material for: Voices from the front line: Community health workers’ perspectives on effectiveness and needs in Madagascar’s tuberculosis response
Source: PLOS Glob Public Health. 2026 Oct 22;6(10):e0005797. doi: 10.1371/journal.pgph.0005797 (PMC13690241; doi:10.1371/journal.pgph.0005797)
Supplement: Supplementary file 5 [file pgph.0005797.s006.docx]

**Response to reviewers’ and editor’s comments**

**Manuscript title:** Voices from the front line: community health workers’ perspectives on effectiveness and needs in Madagascar’s tuberculosis response

**Manuscript ID:** PGPH-D-25-03991

**Date:** June 05, 2026

Dear reviewers, dear editor,

We sincerely thank you for the careful and constructive evaluation and the detailed feedback on our manuscript. We greatly appreciate the time the reviewers invested and recognise the importance of their comments for improving the quality and relevance of our research. We have revised the manuscript thoughtfully and hope the changes substantially strengthen it.

Yours faithfully,

The authors

| **Response to Reviewer #2** |  |
| --- | --- |
| 1. The analytical contribution remains somewhat limited. The five major themes identified are highly consistent with existing CHW literature globally. While the framing around implementation gaps and fragmentation is promising, the manuscript would benefit from deeper analytical engagement explaining how these findings extend beyond already well-established barriers in CHW programs. | We thank the reviewer for this important comment and agree that the analytical contribution warranted further clarification. We have added a corresponding, explicit paragraph at the start of the **Discussion (page 24, lines 498-504)** highlighting the three ways in which our analysis extends current literature:   - it shifts the focus from what support CHWs require to how accountability for that support is operationalised (***“Accountability for essential support”***); - it demonstrates how fragmentation generates inequity within the CHW workforce itself (***“Fragmentation and intra-workforce inequity”***); and - it shows from the workforce's own perspective, that the central tension is less between volunteerism and salaried employment than about credible governance configurations (***“Governance configurations beyond* volunteer-salaried binary*”***).   These three threads are developed in the Discussion paragraphs, including dedicated headings to strengthen the structure of the Discussion.  Of note, we’ve retained the Results section as primarily descriptive to remain consistent with previous feedback. |
| 2. The governance and accountability discussion occasionally goes beyond what is directly supported by the empirical material. Some normative claims regarding rights, accountability, and national stewardship would benefit from closer grounding in participant narratives. | We thank the reviewer for this observation and agree that some formulations in the previous version risked presenting normative claims as standalone arguments rather than as interpretations anchored in participant narratives. We have revised the Discussion at three points as follows:   - ***Discussion (page 25, lines 519-520)****: “CHWs’ consistent emphasis on the gap between promised entitlements and lived support resonates with broader calls to treat CHWs as rights-holders and system actors, while underscoring that accountability must be understood as a condition for sustaining trust and motivation.”* - ***Discussion (page 27, lines 564-567): “****In this context, professionalisation appears less as a contractual shift and more as a governance configuration grounded in reliability, fairness, and recognition that participants identified as preconditions for meaningful engagement.”* - ***Discussion (page 28, lines 575-578):*** *“Reflecting the multi-actor implementation landscape that participants described, formal stewardship rests with the Malagasy government, while both government services and implementing partners shape how consistently and equitably support is delivered in practice.”* |
| 3. The participatory action research framing requires further clarification. It is currently unclear how CHWs participated beyond data generation and recommendation discussions. Please clarify whether participants were involved in interpretation, coding, validation, or dissemination processes | We agree that the previous version of the paper did not clearly indicate the scope of CHWs’ involvement in the participatory action research framing, which we now clarified as follows:   - ***Study design (page 6, lines 124-127):*** *“The findings were subsequently presented at a design-thinking workshop held in March 2025 in Analamanga region, convening CHWs and policy makers, where participants jointly validated the identified priorities and defined actionable measures for strengthening the CHW workforce.”* - ***Data collection (page 10, lines 205-209):*** *“Each discussion concluded with a co-design segment to generate practical, context-based recommendations for strengthening community health implementation. However, CHWs were not involved in data coding, formal analysis, or theme development, which remained researcher-led within a reflexive thematic analysis framework.”* - ***Data analysis (pages 11-12, lines 237-239):*** *“CHWs were not involved in formal coding or theme development; however, interpretive discussions during FGDs contributed to refining emerging understandings and informing practice-oriented recommendations.”* |
| 4. The use of TB as a tracer condition should be theoretically strengthened. The manuscript would benefit from a clearer explanation of why TB specifically provides insight into broader CHW system governance and how transferable these findings are beyond TB programming. | We thank the reviewer for this important comment. We have addressed both points in two places:   - ***Study Design (page 6, lines 128-138):*** *“To anchor the analysis in specific service delivery realities while examining broader system enablers and barriers, we used TB as a tracer condition for two reasons. First, Madagascar’s persistently high TB burden (estimated incidence of 233 cases per 100,000 population, unchanged since 2013 [16]) makes effective community-based TB service delivery a national necessity. Second, TB combines several features that make it analytically demanding and therefore well suited as a tracer of broader CHW system support: it requires sustained, multi-touchpoint engagement (sensitisation, screening, referral, treatment adherence support, and contact tracing); it is highly stigma-sensitive; and it depends on reliable infection prevention measures and equipment. Altogether, these features place high demands on the structural support that characterise CHW programmes, and suggest that weaknesses in any of these dimensions become visible in CHWs’ lived experiences of TB-related work.”* - ***Discussion (page 24, lines 492-497):*** *“We therefore anticipate that findings will be broadly transferable across community health domains in Madagascar, since most programmes share a similar set of structural support needs. However, given the marked fragmentation of community health implementation across actors and financing streams, this transferability requires confirmation through further domain-specific research.”* |
| 5. Although the reflexivity section is appreciated, additional reflection on the influence of institutional affiliations and possible social desirability bias would strengthen the manuscript further. | We thank the reviewer for this valuable suggestion. In response, we enhanced the appropriate sections by adding the following content:  ***Reflexivity and positionality (page 30, lines 624-627):*** *“Participants may have perceived the research team as linked to NGOs, donor-supported programmes, or health institutions, which could have shaped how they expressed criticism or expectations. The team therefore remained attentive to potential social desirability bias and power asymmetries throughout the study.”*  ***Data collection (pages 9-10, lines 193-197):*** *“The research team remained aware that participants might perceive researchers as linked to donor-supported health structures, potentially shaping how they expressed criticism or expectations. To reduce this influence, moderators emphasized the independence of the study and encouraged open discussion of both positive and negative experiences.”*  ***Strengths and limitations*** ***(page 28, lines 594-598)****: “Fifth, participants may have perceived the research team as linked to NGOs, donor-supported programmes, or health institutions, which could have influenced how they expressed criticism or expectations; the team remained attentive to this potential social desirability bias throughout the study.”* |
| 6. Finally, the manuscript would benefit from more careful calibration between the scope of the dataset (26 CHWs across two regions using FGDs only) and the breadth of some national-level conclusions. | We thank the reviewer for this point and agree that the previous **Discussion** contained broader claims than the empirical scope of the dataset warranted. We have now improved the language at multiple points throughout to anchor our interpretations in the experiences of CHWs from the two study regions. Specifically:   - ***Page 23 line 474:*** *"CHWs participating in this study across two regions…" … "..., suggesting that they are systemic rather than context-specific constraints in community health implementation".* - ***Page 26, line 534:*** *“Participants described this as….”.* - ***Page 28, line 574:*** *“Establishing a more effective national CHW programme in similar contexts will likely…”* - ***Page 24, lines 492-497*** *(see also response to comment 4 Rn°2 (aspect 2) for details).*   The dedicated **Strengths and limitations subsection** now explicitly states that *"the study was limited to five FGDs across two regions and may not capture the full diversity of CHW experiences nationwide"* *(****page 28, lines 584-585****).* |
| **Response to Reviewer #5** |  |
| 1. Additional background on TB is needed, including epidemiology, particularly within the region, and an overview of TB program implementation and management in Madagascar. Some of this information appears in the Methods section but would be more appropriately located in the Introduction. | We thank the reviewer for this helpful suggestion. We have added a dedicated paragraph in the Introduction covering national TB epidemiology, undernutrition as a contextual risk factor, the notification gap, regional burden disparities, the structure and financing of the National TB Programme (PNLT), and the role of CHWs within it as follows:  ***Introduction (pages 4-5, lines 81-94):*** *“Tuberculosis (TB) remains a major public health challenge in Madagascar. The estimated TB incidence has remained largely unchanged at 233 cases per 100,000 population since 2013 [15]. Around 40% of children under five years of age are stunted, indicating high rates of undernutrition among children, a major risk factor for developing TB [16]. Estimates indicate that only about half of incident TB cases are notified and treated annually, pointing to substantial gaps in case detection and access to care [17]. The southern regions of the country, including the Grand Sud, bear a disproportionate burden, with notification rates substantially higher than in the central highlands [18]. Madagascar's National TB Programme (Programme National de Lutte contre la Tuberculose, PNLT), established in 1991 and predominantly financed through international donors, coordinates TB care through a country-wide network of public, faith-based, and private diagnostic and treatment centres [17]. CHWs are positioned as the community-level arm of the programme, contributing to sensitisation, presumptive case identification, referral, treatment adherence support, and household contact tracing [14].”*  Regional TB notification rates for the two study regions as well as socio-economic descriptors remain in the Study Setting section, where they describe the immediate study context. We hope this sufficiently addresses this need. |
| 2. Tuberculosis should be written in full at first mention before using the abbreviation “TB.” | We thank the reviewer for this comment and apologize for this oversight. We now write out the word in full at first mention ***(Introduction \| page 4, line 81).*** |
| 3. Please clarify the training, education, and professional background required for CHWs, particularly those working within Madagascar’s TB program. | We thank the reviewer for this helpful comment. We added appropriate information as follows:  ***Study setting (page 7, lines 148-151):*** *“Under national policy, CHWs are volunteer, community-selected lay health workers without formal education requirements; basic literacy and community acceptance are typically considered for selection, and training varies across the Ministry of Public Health and partner-supported programmes. ”* |
| 4. On line 149, the term “registered as CHWs” is used. Please clarify whether CHWs are formally registered health professionals in this context (e.g., similar to nurses or physicians). If not, consider revising this wording. | Thank you for highlighting this need for clarification. We revised the Methods section to clarify the inclusion and exclusion criteria and removed the term “officially registered,” which may be insufficiently precise. We now say:  ***Sampling and selection of participants (page 8-9, lines 173-175):*** *“...were not officially selected by their community based on local practices (including identification by their communities and validation by the head of the selected health centers), …”* |
| 5. It is unclear whether data saturation was achieved. While numerical saturation is mentioned, please specify whether and how data saturation was assessed across study groups. This should be explicitly addressed in the Methods. | We thank the reviewer for this need of methodological clarification. We now better explain how data adequacy was assessed in the context of reflexive thematic analysis:  ***Data analysis (page 12, lines 248-253):*** *“Recurring patterns and priorities were identified across the five FGDs, including among participants from both urban and rural settings. While some contextual differences emerged, particularly regarding access to training and material support, no substantially new thematic dimensions arose in the later discussions. The recurrence of key themes across two contrasting regions supported the sufficiency of the dataset for addressing the study objectives.”* |
| 6. The Methods section is quite lengthy and would benefit from greater conciseness | We thank the reviewer for this suggestion and acknowledge that the Methods section gained length over the review process. We have revised it for conciseness throughout, condensing the **Study setting** paragraph and substantially consolidating the **Data collection** and **Data analysis** sections. |
| 7. Consider including the interview guide as a supplementary file. This would allow for a more streamlined description within the Methods section | We thank the reviewer for this helpful suggestion and now add the FGD guide as **supplementary file 1** to the manuscript (including reference to this supplement on **page 10, lines 202-203**). |
| 8. Consider providing additional demographic information where available, such as education level, training, employment status (paid vs volunteer), and years of experience. If all CHWs are unpaid, this should be clearly stated in the participant characteristics. | We thank the reviewer for this helpful suggestion. We have addressed this in two places in the Methods section:   - ***Study setting (page 7, lines 146-151):*** *“A national network of approximately 50,700 volunteer CHWs serves on the front line [21], providing prevention, screening, and patient support and linking communities to CSBs. Under national policy, CHWs are volunteer, community-selected lay health workers without formal education requirements; basic literacy and community acceptance are typically considered for selection, and training varies across the Ministry of Public Health and partner-supported programmes.”* - ***Sampling and selection of participants (page 9, lines 179-183):*** *“Detailed sociodemographic characteristics such as level of education, years of experience, and training exposure were not systematically collected during data collection. All participating CHWs were volunteers without a formal salary.”* |
| 9. You may consider including a thematic map or figure to illustrate the relationships between themes; however, this is optional. | We thank the reviewer for this thoughtful suggestion. After careful consideration, we opted not to add a separate thematic map at this revision stage. We hope that the clarifications introduced in response to other comments will help readers sufficiently in understanding the relationships between themes. We are grateful for the reviewer's flexibility in noting this as optional. |
| 10. The themes currently appear largely descriptive rather than analytical. In line with Braun and Clarke’s approach to thematic analysis, themes should be interpretive, coherent, and narratively organised, moving beyond description to provide deeper insight into meaning across the dataset. | We thank the reviewer for this methodological observation. In line with Braun and Clarke's reflexive TA framework, we have revised the framing of each of the five themes in the Results section and each theme now opens with an interpretive statement capturing its central meaning across the dataset. For example:   - ***Stable and equitable socio-economic protection (page 13, lines 270-273):*** *"Across both regions, socio-economic protection emerged less as a request for income than as a marker of whether the system supporting CHWs could be trusted. Participants framed reliability, transparency, and equity in distribution as defining features of economic protection."* - ***Recognition and integration into the health system (page 22, lines 446-447):*** *"Recognition was articulated not as a symbolic gesture but as a precondition for legitimacy; both within the formal health system and within the communities CHWs serve."* - The analytical interlinkages between the five themes are further developed in the Discussion, where they are organised around three explicit threads (see also response to Comment 1 Rn°2 & Comment 15 Rn°5). |
| 11. At times, the Results section shifts into discussion-style commentary (e.g., recommendations for reform). These interpretations would be more appropriate in the Discussion section. | We thank the reviewer for this useful comment. We have revised the **Results** section to remove interpretive and reform-oriented statements and to focus on the experiences and perceptions of the participants. Specifically, we removed “to ensure/in order to/so that” clauses and also interpretative statements describing effects. We shifted the corresponding content, if not already mentioned there, to the appropriate discussion section. |
| 12. Some participant quotes are lengthy and could be shortened (e.g., using ellipses “…”) without losing meaning. This would allow space for additional illustrative quotes. | We thank the reviewer for this suggestion. We have reviewed the quotations and shortened selected excerpts where appropriate using ellipses while ensuring that meaning and context are preserved. For some longer quotations we decided to retain them when needed to reflect the complexity of participants’ accounts. |
| 13. It would strengthen the analysis to indicate the prevalence of viewpoints and whether perspectives differed across groups (e.g., urban vs rural CHWs). | We thank the reviewer for this valuable suggestion and revised the **Results** and **Discussion** sections accordingly; we have explicitly marked both shared perspectives across the FGDs and differences emerging between urban, peri-urban, and rural participants, while avoiding over-quantification in line with reflexive TA.   - ***Training (page 16, lines 333–335):*** *"Another recurrent concern was perceived inequity in access to training, with opportunities varying widely by location and implementation partners, disfavouring specifically CHWs in rural and remote areas."* - ***Transport (page 20, lines 415-417):*** *"Transport-related difficulties were particularly emphasized by rural CHWs, who described walking long distances to reach patients or health centers, which they experienced as physically demanding and financially costly.”*   Where perspectives converged across groups, this is now explicitly signalled, e.g., in the opening of the **Discussion (page 23, lines 477-479):** *"These priorities were consistent across the two contrasting regions, suggesting that they are systemic rather than context-specific constraints in community health implementation."*  The **Strengths and limitations** subsection further notes that the recurrence of key themes across two contrasting regions supports the adequacy of the dataset for the study objectives (see response to comment 16). |
| 14. Consider including participant identifiers (e.g., P3 or pseudonyms such as “Jocelyn”) to demonstrate variation in perspectives across quotes. | We thank the reviewer for this suggestion. Within the current manuscript, participant quotations are identified by gender and geographic context (urban, peri-urban or rural setting and region), which we considered analytically relevant to the study objectives and sufficient to reflect variation in perspectives while preserving participant confidentiality.  To improve clarity, we checked the consistency of the quotation identifiers throughout the manuscript. However, pseudonyms and participant codes were not used as the analysis was of contextual patterns rather than individual narratives. |
| 15. The Discussion section is somewhat repetitive and would benefit from improved structure and clearer progression of ideas. | We thank the reviewer for this useful observation. As detailed in our response to Reviewer 2 (comment 1), we have restructured the **Discussion** around three explicit analytical threads, each now under a dedicated subheading. We’ve also revised the whole Discussion and consolidated overlapping content between the previously separate fragmentation- and accountability-paragraphs. We hope this provides a clearer progression of ideas. |
| 16. Consider including dedicated Strengths and Limitations and Conclusion sections, and further elaborating on the study’s limitations. | We thank the reviewer for this helpful suggestion. We have now explicitly framed and extended the appropriate section as follows:  ***Strengths and limitations (page 28-29, lines 581-600):*** *“This study provides systematically analysed qualitative evidence foregrounding CHWs’ perspectives in Madagascar. Strengths include its participatory design embedded into a broader action research initiative, the inclusion of two contrasting regions, the use of local dialects to enable authentic participant expression, and the subsequent validation of findings with CHWs and policymakers through a joint workshop. Several limitations should be considered when interpreting the findings. First, the study was limited to five FGDs across two regions and may not capture the full diversity of CHW experiences nationwide. Second, the use of TB as a tracer condition facilitated concrete discussions of service delivery realities, including stigma, long-term follow-up, infection risk, and referral pathways, but may have emphasized priorities specific to infectious disease programmes and may not be fully generalisable across all community health activities. Third, the gender composition of participants reflects contextual workforce structures in urban and rural settings and may have shaped the perspectives expressed. Fourth, the analysis was led by a single coder, with researcher triangulation at key interpretive moments rather than full double-coding. Fifth, participants may have perceived the research team as linked to NGOs, donor-supported programmes, or health institutions, which could have influenced how they expressed criticism or expectations; the team remained attentive to this potential social desirability bias throughout the study. Despite these limitations, the system-level enablers identified here are consistent with findings from CHW programmes in other settings and were validated through subsequent stakeholder engagement.”* |
