# Supplementary material for: Voices from the front line: Community health workers’ perspectives on effectiveness and needs in Madagascar’s tuberculosis response
Source: PLOS Glob Public Health. 2026 Oct 22;6(10):e0005797. doi: 10.1371/journal.pgph.0005797 (PMC13690241; doi:10.1371/journal.pgph.0005797)
Supplement: Peer review history

# Peer review: Decision Letter 0

**DOI:** 10.1371/journal.pgph.0005797.r001

**Authors:** Md. Alamgir Hossain

1 Feb 2026

PGPH-D-25-03991

Voices from the front line: community health workers’ perspectives on effectiveness and needs in Madagascar’s tuberculosis response

PLOS Global Public Health

Dear Dr. Nadine Muller,

Thank you for submitting your manuscript to PLOS Global Public Health. After careful consideration, we feel that it has merit but does not fully meet PLOS Global Public Health’s publication criteria as it currently stands. Therefore, we invite you to submit a revised version of the manuscript that addresses the points raised during the review process.

Please submit your revised manuscript by . If you will need more time than this to complete your revisions, please reply to this message or contact the journal office at globalpubhealth@plos.org. When you're ready to submit your revision, log on to https://www.editorialmanager.com/pgph/ and select the 'Submissions Needing Revision' folder to locate your manuscript file.

Please include the following items when submitting your revised manuscript:

- A letter that responds to each point raised by the editor and reviewer(s). You should upload this letter as a separate file labeled 'Response to Reviewers'.
- A marked-up copy of your manuscript that highlights changes made to the original version. You should upload this as a separate file labeled 'Revised Manuscript with Track Changes'.
- An unmarked version of your revised paper without tracked changes. You should upload this as a separate file labeled 'Manuscript'.

Guidelines for resubmitting your figure files are available below the reviewer comments at the end of this letter.

We look forward to receiving your revised manuscript.

Kind regards,

Md. Alamgir Hossain, M.S.S., M.P.H.

Academic Editor

PLOS Global Public Health

Journal Requirements:

1. Please ensure that your Ethics Statement is available in its entirety at the beginning of your Methods section, under a subheading 'Ethics Statement'.

2. We have noticed that you have uploaded Supporting Information files, but you have not included a list of legends. Please add a full list of legends for your Supporting Information files after the references list.

3. In the online submission form, you indicated that “The data supporting this study are not publicly available due to ethical restrictions. Interested researchers may request access to the data by contacting the corresponding author.”

All PLOS journals now require all data underlying the findings described in their manuscript to be freely available to other researchers, either

1. In a public repository,

2. Within the manuscript itself, or

3. Uploaded as supplementary information.

This policy applies to all data except where public deposition would breach compliance with the protocol approved by your research ethics board. If your data cannot be made publicly available for ethical or legal reasons (e.g., public availability would compromise patient privacy), please explain your reasons by return email and your exemption request will be escalated to the editor for approval. Your exemption request will be handled independently and will not hold up the peer review process, but will need to be resolved should your manuscript be accepted for publication. One of the Editorial team will then be in touch if there are any issues.

If the reviewer comments include a recommendation to cite specific previously published works, please review and evaluate these publications to determine whether they are relevant and should be cited. There is no requirement to cite these works unless the editor has indicated otherwise.

Additional Editor Comments (if provided):

[Note: HTML markup is below. Please do not edit.]

Reviewers' comments:

Reviewer's Responses to Questions

**Comments to the Author**

1. Does this manuscript meet PLOS Global Public Health’s publication criteria?

Reviewer #1: Yes

Reviewer #2: No

**********

2. Has the statistical analysis been performed appropriately and rigorously?

Reviewer #1: N/A

Reviewer #2: No

**********

3. Have the authors made all data underlying the findings in their manuscript fully available (please refer to the Data Availability Statement at the start of the manuscript PDF file)?

The PLOS Data policy

Reviewer #1: No

Reviewer #2: No

**********

4. Is the manuscript presented in an intelligible fashion and written in standard English?

Reviewer #1: Yes

Reviewer #2: No

**********

Reviewer #1: Thank you for a very interesting and valuable contribution on CHW, especially focusing on availability, accessibility, acceptability, and quality of health services and the essential conceptual shifting of CHW from assets to rights-holders.

The paper presents a compelling narrative from FGDs with CHWs that highlights key aspects to promote UHC. Using Tuberculosis (TB) programme support as a tracer condition is also a commendable approach. The paper has the potential with some restructuring and edits to make a important contribution to the journal.

With this said, I have a number of comments for each section.

Introduction

I lack a clearly stated research aim or question. The closest I found was what was mentioned Line 87-88 that the "research initiative aimed at strengthening Madagascar’s community health workforce". This is furthermore quite a broad and not very specific aim. I would thus suggest for the authors to more clearly state the papers´ aim.

Methods

The study design and study setting is well detailed.

Line 121- 127. I get a sense of the inclusion criteria, where there also exclusion criteria. Maybe state these more explicitly.

Line 140-143. How, where and with whom were the thematic guide pilot-tested?

Line 147.How was written and oral informed consent gathered? Where participants informed about the aims of the study, the data collection and analysis process and potential benefits of the study?

Line 152. Please motivate the choice of analysis approach and I would suggest adding some references to support the choice of analysis.

Line 154-160 Perhaps a table or illustration would help the reader in grasping the analysis process.

Line 161- 165 Clarify the motivation for focusing the analysis with the first author and only having a second author coming in to review specific excerpts and codes.

Line 176-177 As mentioned regarding line 147 clarify the process through which this oral and written consent was obtained.

Results

The results are overall well detailed with excerpts of participants discussions and connecting narrative highlighting the key themes. However, recurring sentences seem to go further into the authors reflective analysis which makes it a bit more difficult what is the results of the discussions and originates from the participants discussions and what is the authors interpretations. I have included a number of examples of this below.

Line 203-206, This has parts that are more analytical reflection and perhaps would be better suited for the discussion section, leaving the results section more focused on reporting. Its a good reflection just unsure its placement is right.

Line 218-219, This sentence also seem to more belong in the discussion.

Line 221-223 This is another sentence that could work better in the discussion.

Line 246-248, Consider moving to discussion.

Line 250- onward. This theme is much better in focusing on reporting on what the participants said and discussed without adding further analytical framing that more belongs to the discussion.

Line 286-287. I suggest to be clear in the wording, likely participants expressed perceptions as part of the FGDs.

Line 324-325. Clarify if this is what was expressed by the participants or the authors analysis.

Line 328-330. Please be clear if this is what was expressed by the participants.

Line 341-342. Maybe be clear that we only know that its what CHWs expressed rather than what they did.

Line 349-351. I would suggest formulating it as “CHWs expressed how they often…”

Line 368. Clarify that this is what was expressed.

Line 398. Maybe clarify that it is what was expressed by the participants.

Discussion

The discussion is overall well-presented and raises multiple important points and contributions of the paper, as exemplified in Line 418-423 where analytical reflection is very relevant. However, I have some minor comments.

Line 410-411. Maybe reference what research has been done on CHW´s in Madagascar if any.

While UHC was outlined as the key focus with TB as a tracer condition programme support its only directly mention and engaged with on two points in the discussion, line 409 and 495. I would suggest that the authors more actively engage with UHC in the discussion.

Data availability

Please refer to the journal’s requirements of data availability.

Reviewer #2: This manuscript addresses an important topic regarding community health workers and TB care in Madagascar and is clearly written with strong policy framing. However, significant methodological and analytical weaknesses limit its suitability for publication in its current form.

Key Concerns

1. Insufficient Qualitative Rigor

The manuscript does not adequately demonstrate data saturation, coding procedures, reflexivity, or analytical validation. The methodological transparency expected for qualitative research is lacking.

2. Descriptive Rather Than Analytical Findings

The themes presented (training, supervision, payment, tools, recognition) are well-known in CHW literature and are described rather than deeply analyzed.

3. TB Not Effectively Used as a Tracer Condition

Although TB is introduced as a tracer for UHC, the findings are not TB-specific and do not analytically leverage this framing.

4. Discussion Overreaches the Data

Governance and policy critiques in the discussion are not sufficiently grounded in the empirical findings.

5. Data Availability Does Not Meet PLOS Standards

“Available upon request” is insufficient and non-compliant with PLOS data-sharing policy.

6. Limited Novel Contribution

The study offers contextual insight but little new conceptual or theoretical contribution.

**********

what does this mean?). If published, this will include your full peer review and any attached files.

**Do you want your identity to be public for this peer review?**  If you choose “no”, your identity will remain anonymous but your review may still be made public.

For information about this choice, including consent withdrawal, please see our Privacy Policy

Reviewer #1: **Yes:**  Martin Mickelsson

Reviewer #2: No

**********

[NOTE: If reviewer comments were submitted as an attachment file, they will be attached to this email and accessible via the submission site. Please log into your account, locate the manuscript record, and check for the action link "View Attachments". If this link does not appear, there are no attachment files.]

# Author comment: Author response to Decision Letter 1

**DOI:** 10.1371/journal.pgph.0005797.r002

3 Mar 2026

Attachment Submitted filename: CHW_Voices_Response to Reviewers_FV_23022026.pdf

# Peer review: Decision Letter 1

**DOI:** 10.1371/journal.pgph.0005797.r003

**Authors:** Md. Alamgir Hossain

13 Apr 2026

PGPH-D-25-03991R1

Voices from the front line: community health workers’ perspectives on effectiveness and needs in Madagascar’s tuberculosis response

PLOS Global Public Health

Dear Dr. Muller,

Thank you for submitting your manuscript to PLOS Global Public Health. After careful consideration, we feel that it has merit but does not fully meet PLOS Global Public Health’s publication criteria as it currently stands. Therefore, we invite you to submit a revised version of the manuscript that addresses the points raised during the review process.

Please submit your revised manuscript by 10/05/2026. If you will need more time than this to complete your revisions, please reply to this message or contact the journal office at globalpubhealth@plos.org. When you're ready to submit your revision, log on to https://www.editorialmanager.com/pgph/ and select the 'Submissions Needing Revision' folder to locate your manuscript file.

Please include the following items when submitting your revised manuscript:

- A letter that responds to each point raised by the editor and reviewer(s). You should upload this letter as a separate file labeled 'Response to Reviewers'.
- A marked-up copy of your manuscript that highlights changes made to the original version. You should upload this as a separate file labeled 'Revised Manuscript with Track Changes'.
- An unmarked version of your revised paper without tracked changes. You should upload this as a separate file labeled 'Manuscript'.

Guidelines for resubmitting your figure files are available below the reviewer comments at the end of this letter.

As the corresponding author, your ORCID iD is verified in the submission system and will appear in the published article. PLOS supports the use of ORCID, and we encourage all coauthors to register for an ORCID iD and use it as well. Please encourage your coauthors to verify their ORCID iD within the submission system before final acceptance, as unverified ORCID iDs will not appear in the published article. *Only*  the individual author can complete the verification step; PLOS staff *cannot*  verify ORCID iDs on behalf of authors.

We look forward to receiving your revised manuscript.

Kind regards,

Md. Alamgir Hossain, M.S.S., M.P.H.

Academic Editor

PLOS Global Public Health

Journal Requirements:

If the reviewer comments include a recommendation to cite specific previously published works, please review and evaluate these publications to determine whether they are relevant and should be cited. There is no requirement to cite these works unless the editor has indicated otherwise.

Please review your reference list to ensure that it is complete and correct. If you have cited papers that have been retracted, please include the rationale for doing so in the manuscript text, or remove these references and replace them with relevant current references. Any changes to the reference list should be mentioned in the rebuttal letter that accompanies your revised manuscript. If you need to cite a retracted article, indicate the article’s retracted status in the References list and also include a citation and full reference for the retraction notice.

Additional Editor Comments (if provided):

[Note: HTML markup is below. Please do not edit.]

Reviewers' comments:

Reviewer's Responses to Questions

**Comments to the Author**

Reviewer #3: All comments have been addressed

Reviewer #4: (No Response)

**********

publication criteria?

Reviewer #3: Yes

Reviewer #4: Yes

**********

3. Has the statistical analysis been performed appropriately and rigorously?

Reviewer #3: N/A

Reviewer #4: N/A

**********

4. Have the authors made all data underlying the findings in their manuscript fully available (please refer to the Data Availability Statement at the start of the manuscript PDF file)?

The PLOS Data policy

Reviewer #3: Yes

Reviewer #4: Yes

**********

5. Is the manuscript presented in an intelligible fashion and written in standard English?

Reviewer #3: Yes

Reviewer #4: Yes

**********

Reviewer #3: Please ensure that you include your reflexivity statement - it does not appear to be in either the clean or tracked version at the lines you listed.

Reviewer #4: I note that the included respondents are female dominated in one site, and male dominated in the other. Please include in your methodology description the extent to which this reflects the overall situation of active CHWs in the two sites. Also discuss in your limitations the possible influence of this to the study results.

It may be useful to include a sex-based approach in analysis of the results, to reflect any differences in views expressed by females and males.

**********

what does this mean?). If published, this will include your full peer review and any attached files.

**Do you want your identity to be public for this peer review?**  If you choose “no”, your identity will remain anonymous but your review may still be made public.

For information about this choice, including consent withdrawal, please see our Privacy Policy

Reviewer #3: No

Reviewer #4: No

**********

[NOTE: If reviewer comments were submitted as an attachment file, they will be attached to this email and accessible via the submission site. Please log into your account, locate the manuscript record, and check for the action link "View Attachments". If this link does not appear, there are no attachment files.]

# Author comment: Author response to Decision Letter 2

**DOI:** 10.1371/journal.pgph.0005797.r004

17 Apr 2026

Attachment Submitted filename: Response to reviewers_R2.docx

# Peer review: Decision Letter 2

**DOI:** 10.1371/journal.pgph.0005797.r005

**Authors:** Md. Alamgir Hossain

8 May 2026

PGPH-D-25-03991R2

Voices from the front line: community health workers’ perspectives on effectiveness and needs in Madagascar’s tuberculosis response

PLOS Global Public Health

Dear Dr. Muller,

Thank you for submitting your manuscript to PLOS Global Public Health. After careful consideration, we feel that it has merit but does not fully meet PLOS Global Public Health’s publication criteria as it currently stands. Therefore, we invite you to submit a revised version of the manuscript that addresses the points raised during the review process.

Please submit your revised manuscript by 15/06/2026. If you will need more time than this to complete your revisions, please reply to this message or contact the journal office at globalpubhealth@plos.org. When you're ready to submit your revision, log on to https://www.editorialmanager.com/pgph/ and select the 'Submissions Needing Revision' folder to locate your manuscript file.

Please include the following items when submitting your revised manuscript:

- A letter that responds to each point raised by the editor and reviewer(s). You should upload this letter as a separate file labeled 'Response to Reviewers'.
- A marked-up copy of your manuscript that highlights changes made to the original version. You should upload this as a separate file labeled 'Revised Manuscript with Track Changes'.
- An unmarked version of your revised paper without tracked changes. You should upload this as a separate file labeled 'Manuscript'.

Guidelines for resubmitting your figure files are available below the reviewer comments at the end of this letter.

As the corresponding author, your ORCID iD is verified in the submission system and will appear in the published article. PLOS supports the use of ORCID, and we encourage all coauthors to register for an ORCID iD and use it as well. Please encourage your coauthors to verify their ORCID iD within the submission system before final acceptance, as unverified ORCID iDs will not appear in the published article. *Only*  the individual author can complete the verification step; PLOS staff *cannot*  verify ORCID iDs on behalf of authors.

We look forward to receiving your revised manuscript.

Kind regards,

Md. Alamgir Hossain, M.S.S., M.P.H.

Academic Editor

PLOS Global Public Health

**Journal Requirements:**

If the reviewer comments include a recommendation to cite specific previously published works, please review and evaluate these publications to determine whether they are relevant and should be cited. There is no requirement to cite these works unless the editor has indicated otherwise.

**Additional Editor Comments (if provided):**

[Note: HTML markup is below. Please do not edit.]

Reviewers' comments:

Reviewer's Responses to Questions

**Comments to the Author**

Reviewer #2: All comments have been addressed

Reviewer #5: (No Response)

**********

publication criteria?

Reviewer #2: Partly

Reviewer #5: Yes

**********

3. Has the statistical analysis been performed appropriately and rigorously?

Reviewer #2: Yes

Reviewer #5: Yes

**********

4. Have the authors made all data underlying the findings in their manuscript fully available (please refer to the Data Availability Statement at the start of the manuscript PDF file)?

The PLOS Data policy

Reviewer #2: Yes

Reviewer #5: Yes

**********

5. Is the manuscript presented in an intelligible fashion and written in standard English?

Reviewer #2: No

Reviewer #5: Yes

**********

**Reviewer #2:**  Thank you for the opportunity to review this important and thoughtfully revised manuscript. The paper addresses a highly relevant topic for global public health and community health systems strengthening. The manuscript is clearly written, and the inclusion of reflexivity, positionality, and methodological clarification has strengthened the paper considerably.

I nevertheless have several concerns that should be addressed before publication:

1. The analytical contribution remains somewhat limited. The five major themes identified are highly consistent with existing CHW literature globally. While the framing around implementation gaps and fragmentation is promising, the manuscript would benefit from deeper analytical engagement explaining how these findings extend beyond already well-established barriers in CHW programs.

2. The governance and accountability discussion occasionally goes beyond what is directly supported by the empirical material. Some normative claims regarding rights, accountability, and national stewardship would benefit from closer grounding in participant narratives.

3. The participatory action research framing requires further clarification. It is currently unclear how CHWs participated beyond data generation and recommendation discussions. Please clarify whether participants were involved in interpretation, coding, validation, or dissemination processes.

4. The use of TB as a tracer condition should be theoretically strengthened. The manuscript would benefit from a clearer explanation of why TB specifically provides insight into broader CHW system governance and how transferable these findings are beyond TB programming.

5. Although the reflexivity section is appreciated, additional reflection on the influence of institutional affiliations and possible social desirability bias would strengthen the manuscript further.

6. Finally, the manuscript would benefit from more careful calibration between the scope of the dataset (26 CHWs across two regions using FGDs only) and the breadth of some national-level conclusions.

**Reviewer #5:**  Thank you for the opportunity to review this manuscript. This study highlights the important role of community health workers (CHWs) and explores their perceptions and experiences within the tuberculosis (TB) program in Madagascar. The identification of key barriers and facilitators is valuable. I have provided comments and recommendations below to help strengthen the manuscript.

Introduction

1. Additional background on TB is needed, including epidemiology, particularly within the region, and an overview of TB program implementation and management in Madagascar. Some of this information appears in the Methods section but would be more appropriately located in the Introduction.

2. Tuberculosis should be written in full at first mention before using the abbreviation “TB.”

3. Please clarify the training, education, and professional background required for CHWs, particularly those working within Madagascar’s TB program.

Methods

4. On line 149, the term “registered as CHWs” is used. Please clarify whether CHWs are formally registered health professionals in this context (e.g., similar to nurses or physicians). If not, consider revising this wording.

5. It is unclear whether data saturation was achieved. While numerical saturation is mentioned, please specify whether and how data saturation was assessed across study groups. This should be explicitly addressed in the Methods.

6. The Methods section is quite lengthy and would benefit from greater conciseness.

7. Consider including the interview guide as a supplementary file. This would allow for a more streamlined description within the Methods section.

Results

8. Consider providing additional demographic information where available, such as education level, training, employment status (paid vs volunteer), and years of experience. If all CHWs are unpaid, this should be clearly stated in the participant characteristics.

9. You may consider including a thematic map or figure to illustrate the relationships between themes; however, this is optional.

10. The themes currently appear largely descriptive rather than analytical. In line with Braun and Clarke’s approach to thematic analysis, themes should be interpretive, coherent, and narratively organised, moving beyond description to provide deeper insight into meaning across the dataset.

11. At times, the Results section shifts into discussion-style commentary (e.g., recommendations for reform). These interpretations would be more appropriate in the Discussion section.

12. Some participant quotes are lengthy and could be shortened (e.g., using ellipses “…”) without losing meaning. This would allow space for additional illustrative quotes.

13. It would strengthen the analysis to indicate the prevalence of viewpoints and whether perspectives differed across groups (e.g., urban vs rural CHWs).

14. Consider including participant identifiers (e.g., P3 or pseudonyms such as “Jocelyn”) to demonstrate variation in perspectives across quotes.

Discussion

15. The Discussion section is somewhat repetitive and would benefit from improved structure and clearer progression of ideas.

16. Consider including dedicated Strengths and Limitations and Conclusion sections, and further elaborating on the study’s limitations.

**********

what does this mean?). If published, this will include your full peer review and any attached files.

**Do you want your identity to be public for this peer review?**  If you choose “no”, your identity will remain anonymous but your review may still be made public.

For information about this choice, including consent withdrawal, please see our Privacy Policy

Reviewer #2: **Yes:**  Somayeh Hessam

Reviewer #5: **Yes:**  Matthew N Berger

**********

[NOTE: If reviewer comments were submitted as an attachment file, they will be attached to this email and accessible via the submission site. Please log into your account, locate the manuscript record, and check for the action link "View Attachments". If this link does not appear, there are no attachment files.]

**Figure Resubmissions** :

# Author comment: Author response to Decision Letter 3

**DOI:** 10.1371/journal.pgph.0005797.r006

10 Jun 2026

Attachment Submitted filename: CHW_Voices_Response to Reviewers_05062026.docx

# Editor report: Decision Letter 3

**DOI:** 10.1371/journal.pgph.0005797.r007

**Authors:** Md. Alamgir Hossain

21 Jul 2026

PGPH-D-25-03991R3

Voices from the front line: community health workers’ perspectives on effectiveness and needs in Madagascar’s tuberculosis response

PLOS Global Public Health

Dear Dr. Nadine Muller,

Thank you for submitting your manuscript to PLOS Global Public Health. After careful consideration, we feel that it has merit but does not fully meet PLOS Global Public Health’s publication criteria as it currently stands. Therefore, we invite you to submit a revised version of the manuscript that addresses the points raised during the review process.

Please submit your revised manuscript by 30/08/2026. If you will need more time than this to complete your revisions, please reply to this message or contact the journal office at globalpubhealth@plos.org. When you're ready to submit your revision, log on to https://www.editorialmanager.com/pgph/ and select the 'Submissions Needing Revision' folder to locate your manuscript file.

Please include the following items when submitting your revised manuscript:

- A letter that responds to each point raised by the editor and reviewer(s). You should upload this letter as a separate file labeled 'Response to Reviewers'.
- A marked-up copy of your manuscript that highlights changes made to the original version. You should upload this as a separate file labeled 'Revised Manuscript with Track Changes'.
- An unmarked version of your revised paper without tracked changes. You should upload this as a separate file labeled 'Manuscript'.

Guidelines for resubmitting your figure files are available below the reviewer comments at the end of this letter.

As the corresponding author, your ORCID iD is verified in the submission system and will appear in the published article. PLOS supports the use of ORCID, and we encourage all coauthors to register for an ORCID iD and use it as well. Please encourage your coauthors to verify their ORCID iD within the submission system before final acceptance, as unverified ORCID iDs will not appear in the published article. *Only*  the individual author can complete the verification step; PLOS staff *cannot*  verify ORCID iDs on behalf of authors.

We look forward to receiving your revised manuscript.

Kind regards,

Md. Alamgir Hossain, M.S.S., M.P.H.

Academic Editor

PLOS Global Public Health

[Note: HTML markup is below. Please do not edit.]

Reviewers' comments:

Reviewer's Responses to Questions

**Comments to the Author**

Reviewer #2: All comments have been addressed

Reviewer #5: (No Response)

**********

publication criteria?

Reviewer #2: Partly

Reviewer #5: Yes

**********

3. Has the statistical analysis been performed appropriately and rigorously?

Reviewer #2: Yes

Reviewer #5: Yes

**********

4. Have the authors made all data underlying the findings in their manuscript fully available (please refer to the Data Availability Statement at the start of the manuscript PDF file)?

The PLOS Data policy

Reviewer #2: Yes

Reviewer #5: Yes

**********

5. Is the manuscript presented in an intelligible fashion and written in standard English?

Reviewer #2: No

Reviewer #5: Yes

**********

**Reviewer #2:**  Thank you for the opportunity to review this important and thoughtfully revised manuscript. The paper addresses a highly relevant topic for global public health and community health systems strengthening. The manuscript is clearly written, and the inclusion of reflexivity, positionality, and methodological clarification has strengthened the paper considerably.

I nevertheless have several concerns that should be addressed before publication:

1. The analytical contribution remains somewhat limited. The five major themes identified are highly consistent with existing CHW literature globally. While the framing around implementation gaps and fragmentation is promising, the manuscript would benefit from deeper analytical engagement explaining how these findings extend beyond already well-established barriers in CHW programs.

2. The governance and accountability discussion occasionally goes beyond what is directly supported by the empirical material. Some normative claims regarding rights, accountability, and national stewardship would benefit from closer grounding in participant narratives.

3. The participatory action research framing requires further clarification. It is currently unclear how CHWs participated beyond data generation and recommendation discussions. Please clarify whether participants were involved in interpretation, coding, validation, or dissemination processes.

4. The use of TB as a tracer condition should be theoretically strengthened. The manuscript would benefit from a clearer explanation of why TB specifically provides insight into broader CHW system governance and how transferable these findings are beyond TB programming.

5. Although the reflexivity section is appreciated, additional reflection on the influence of institutional affiliations and possible social desirability bias would strengthen the manuscript further.

6. Finally, the manuscript would benefit from more careful calibration between the scope of the dataset (26 CHWs across two regions using FGDs only) and the breadth of some national-level conclusions.

**Reviewer #5:**  Thank you for the opportunity to review this manuscript. This study highlights the important role of community health workers (CHWs) and explores their perceptions and experiences within the tuberculosis (TB) program in Madagascar. The identification of key barriers and facilitators is valuable. I have provided comments and recommendations below to help strengthen the manuscript.

Introduction

1. Additional background on TB is needed, including epidemiology, particularly within the region, and an overview of TB program implementation and management in Madagascar. Some of this information appears in the Methods section but would be more appropriately located in the Introduction.

2. Tuberculosis should be written in full at first mention before using the abbreviation “TB.”

3. Please clarify the training, education, and professional background required for CHWs, particularly those working within Madagascar’s TB program.

Methods

4. On line 149, the term “registered as CHWs” is used. Please clarify whether CHWs are formally registered health professionals in this context (e.g., similar to nurses or physicians). If not, consider revising this wording.

5. It is unclear whether data saturation was achieved. While numerical saturation is mentioned, please specify whether and how data saturation was assessed across study groups. This should be explicitly addressed in the Methods.

6. The Methods section is quite lengthy and would benefit from greater conciseness.

7. Consider including the interview guide as a supplementary file. This would allow for a more streamlined description within the Methods section.

Results

8. Consider providing additional demographic information where available, such as education level, training, employment status (paid vs volunteer), and years of experience. If all CHWs are unpaid, this should be clearly stated in the participant characteristics.

9. You may consider including a thematic map or figure to illustrate the relationships between themes; however, this is optional.

10. The themes currently appear largely descriptive rather than analytical. In line with Braun and Clarke’s approach to thematic analysis, themes should be interpretive, coherent, and narratively organised, moving beyond description to provide deeper insight into meaning across the dataset.

11. At times, the Results section shifts into discussion-style commentary (e.g., recommendations for reform). These interpretations would be more appropriate in the Discussion section.

12. Some participant quotes are lengthy and could be shortened (e.g., using ellipses “…”) without losing meaning. This would allow space for additional illustrative quotes.

13. It would strengthen the analysis to indicate the prevalence of viewpoints and whether perspectives differed across groups (e.g., urban vs rural CHWs).

14. Consider including participant identifiers (e.g., P3 or pseudonyms such as “Jocelyn”) to demonstrate variation in perspectives across quotes.

Discussion

15. The Discussion section is somewhat repetitive and would benefit from improved structure and clearer progression of ideas.

16. Consider including dedicated Strengths and Limitations and Conclusion sections, and further elaborating on the study’s limitations.

**********

what does this mean?). If published, this will include your full peer review and any attached files.

**Do you want your identity to be public for this peer review?**  If you choose “no”, your identity will remain anonymous but your review may still be made public.

For information about this choice, including consent withdrawal, please see our Privacy Policy

Reviewer #2: **Yes:**  Somayeh Hessam

Reviewer #5: **Yes:**  Matthew N Berger

# Author comment: Author response to Decision Letter 4

**DOI:** 10.1371/journal.pgph.0005797.r008

29 Jul 2026

Attachment Submitted filename: CHW_Voices_Response_to_Reviewers_05062026_auresp_4.docx

# Peer review: Decision Letter 4

**DOI:** 10.1371/journal.pgph.0005797.r009

**Authors:** Md. Alamgir Hossain

27 Sep 2026

Voices from the front line: community health workers’ perspectives on effectiveness and needs in Madagascar’s tuberculosis response

PGPH-D-25-03991R4

Dear Dr. Nadine Muller,

We are pleased to inform you that your manuscript 'Voices from the front line: community health workers’ perspectives on effectiveness and needs in Madagascar’s tuberculosis response' has been provisionally accepted for publication in PLOS Global Public Health.

Before your manuscript can be formally accepted you will need to complete some formatting changes, which you will receive in a follow up email. A member of our team will be in touch with a set of requests.

Please note that your manuscript will not be scheduled for publication until you have made the required changes, so a swift response is appreciated.

IMPORTANT: The editorial review process is now complete. PLOS will only permit corrections to spelling, formatting or significant scientific errors from this point onwards. Requests for major changes, or any which affect the scientific understanding of your work, will cause delays to the publication date of your manuscript.

If your institution or institutions have a press office, please notify them about your upcoming paper to help maximize its impact. If they'll be preparing press materials, please inform our press team as soon as possible -- no later than 48 hours after receiving the formal acceptance. Your manuscript will remain under strict press embargo until 2 pm Eastern Time on the date of publication. For more information, please contact globalpubhealth@plos.org.

Thank you again for supporting Open Access publishing; we are looking forward to publishing your work in PLOS Global Public Health.

Best regards,

Md. Alamgir Hossain, M.S.S., M.P.H.

Academic Editor

PLOS Global Public Health

***********************************************************

Reviewer Comments (if any, and for reference):

Reviewer's Responses to Questions

**Comments to the Author**

Reviewer #5: All comments have been addressed

Reviewer #6: All comments have been addressed

**********

publication criteria?

Reviewer #5: Yes

Reviewer #6: Yes

**********

3. Has the statistical analysis been performed appropriately and rigorously?

Reviewer #5: N/A

Reviewer #6: Yes

**********

4. Have the authors made all data underlying the findings in their manuscript fully available (please refer to the Data Availability Statement at the start of the manuscript PDF file)?

The PLOS Data policy

Reviewer #5: Yes

Reviewer #6: Yes

**********

5. Is the manuscript presented in an intelligible fashion and written in standard English?

Reviewer #5: Yes

Reviewer #6: Yes

**********

Reviewer #5: The authors have satisfactorily addressed all of my comments. I congratulate them on their work.

Reviewer #6: (No Response)

**********

what does this mean?). If published, this will include your full peer review and any attached files.

**Do you want your identity to be public for this peer review?**  If you choose “no”, your identity will remain anonymous but your review may still be made public.

For information about this choice, including consent withdrawal, please see our Privacy Policy

Reviewer #5: **Yes:**  Matthew N Berger

Reviewer #6: **Yes:**  Dr. Anil Fastenau

**********
